# Motor training‐related brain reorganization in patients with cerebellar degeneration

**DOI:** 10.1002/hbm.25746

**Published:** 2021-12-11

**Authors:** Rossitza Draganova, Frank Konietschke, Katharina M. Steiner, Naveen Elangovan, Meltem Gümüs, Sophia M. Göricke, Thomas M. Ernst, Andreas Deistung, Thilo van Eimeren, Jürgen Konczak, Dagmar Timmann

**Affiliations:** ^1^ Department of Neurology and Center for Translational Neuro‐ and Behavioral Sciences (C‐TNBS) University Hospital Essen, University of Duisburg‐Essen Essen Germany; ^2^ Institute of Biometry and Clinical Epidemiology Charité‐Universitätsmedizin Berlin, Corporate Member of Freie Universität Berlin, Humboldt‐Universität zu Berlin, and Berlin Institute of Health (BIH) Berlin Germany; ^3^ School of Kinesiology University of Minnesota Minneapolis Minnesota USA; ^4^ Department of Neurosurgery University Hospital Essen, University of Duisburg‐Essen Essen Germany; ^5^ Institute for Diagnostic and Interventional Radiology and Neuroradiology University Hospital Essen, University of Duisburg‐Essen Essen Germany; ^6^ Department for Radiation Medicine University Clinic and Outpatient Clinic for Radiology, University Hospital Halle (Saale) Halle (Saale) Germany; ^7^ Multimodal Neuroimaging Group, Department of Nuclear Medicine University of Cologne Cologne Germany

**Keywords:** cerebellar ataxia, motor learning, physical therapy, plasticity, rehabilitation

## Abstract

Cerebellar degeneration progressively impairs motor function. Recent research showed that cerebellar patients can improve motor performance with practice, but the optimal feedback type (visual, proprioceptive, verbal) for such learning and the underlying neuroplastic changes are unknown. Here, patients with cerebellar degeneration (*N* = 40) and age‐ and sex‐matched healthy controls (*N* = 40) practiced single‐joint, goal‐directed forearm movements for 5 days. Cerebellar patients improved performance during visuomotor practice, but a training focusing on either proprioceptive feedback, or explicit verbal feedback and instruction did not show additional benefits. Voxel‐based morphometry revealed that after training gray matter volume (GMV) was increased prominently in the visual association cortices of controls, whereas cerebellar patients exhibited GMV increase predominantly in premotor cortex. The premotor cortex as a recipient of cerebellar efferents appears to be an important hub in compensatory remodeling following damage of the cerebro‐cerebellar motor system.

## Graphical abstract

Voxel‐based morphometry analysis was used to obtain information on the neural correlates of visuomotor arm training in cerebellar degeneration. Whereas controls showed increased gray matter volumes in visual associative cortex, in cerebellar patients gray matter increased in premotor cortex. Findings suggest that the premotor cortex is at center stage for compensatory brain remodeling in patients with cerebellar degeneration, that is a motor area which has well‐known anatomical connections with the cerebellum.

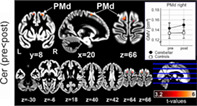

## 1 INTRODUCTION

Cerebellar ataxia affects the coordination and control of gait, posture, upper limb movements, oculomotor function and speech. Ataxia results from focal lesions, such as a stroke, or from a progressive neurodegenerative process. While patients with cerebellar stroke frequently show a good recovery, degenerative cerebellar disease leads to a progressive loss of motor function. Despite several attempts, no drug treatment is currently available that ameliorates the symptoms of cerebellar ataxia (Ilg et al., 2014). Noninvasive and invasive brain stimulation methods have gained interest, but robust and reproducible effects of improving motor function have not been shown (Benussi et al., 2018; Hulst et al., 2017; Miterko et al., 2019). Antisense oligonucleotide therapy may be available in the near future, but will apply only for a subset of trinucleotide repeat disorders (Scoles & Pulst, 2019). Currently, available treatment consists mainly of physical therapy (Ilg et al., 2014; Ilg & Timmann, 2012), but it has been questioned whether such therapy is a useful treatment given that the cerebellum itself is essential for implicit motor learning and such learning becomes impaired in cerebellar disease (Bastian, 2006; Saywell & Taylor, 2008; Thach & Bastian, 2004). However, recent evidence documented that motor training can improve motor function in patients with cerebellar degeneration (Burciu et al., 2013; Ilg et al., 2009; Ilg et al., 2012; Keller & Bastian, 2014; Miyai et al., 2012). Yet, there is still a lack of physical rehabilitation training programs that take knowledge about cerebellar pathophysiology and the opportunities afforded by residual sensorimotor function into account.

With respect to training, it is well documented that sensorimotor learning involves the synergistic engagement of explicit and implicit learning processes (Taylor & Ivry, 2014; Taylor, Krakauer, & Ivry, 2014). Explicit learning is often equated with strategic learning. There is some evidence that cerebellar patients can make use of learning strategies during visuomotor reach adaptation (Taylor, Klemfuss, & Ivry, 2010). However, it is unknown, if additional explicit verbal feedback about movement errors and instruction on how to control for them may aid learning of a sensorimotor skill in patients with cerebellar degeneration. If cerebellar patients could indeed benefit from explicit verbal error feedback or knowledge‐of‐results during training, conventional physical therapy may incorporate this approach to yield better results.

Another aspect of motor learning, which has received little attention in the rehabilitation of degenerative ataxias, relates to the role of proprioception. Given the fact that the cerebellum receives massive proprioceptive afferents through the spinocerebellar tracts (Bloedel, 1973), and given the vital role of proprioceptive information for motor control, it becomes plausible that a training scheme with a focus on the proprioceptive cues could be of help for cerebellar patients (Aman, Elangovan, Yeh, & Konczak, 2015; Saywell & Taylor, 2008). There are reports that proprioception remains intact as cerebellar patients do not show abnormalities in passive position sense tasks, where the limb is passively moved (Bhanpuri, Okamura, & Bastian, 2012; Maschke, Gomez, Tuite, & Konczak, 2003). However, active position sense during voluntary movement becomes impaired in cerebellar degeneration (Bhanpuri et al., 2012), casting doubts, whether a proprioceptive‐focused learning is still possible in patients presenting with degenerative ataxia. Thus, it remains an open question, if these patients can still effectively make use of proprioceptive information to guide motor learning. Finally, and equally important, the underlying neuroplastic changes during training associated with residual learning or compensatory forms of motor learning are only incompletely understood in patients with cerebellar degeneration.

To address these knowledge gaps, we designed a training regimen for a group of people with degenerative ataxia. The main goals of this study were threefold: First, to investigate if the effects of visuomotor training can be enhanced by providing additional explicit motor performance feedback. Second, to gain an understanding if the ability to use proprioceptive error feedback during motor learning is still intact in people with cerebellar degeneration. To that effect, we exposed patients to a training regimen without vision that purely relied on proprioceptive feedback. Third, to delineate the possible neuroplastic changes associated with such learning. We used neuroimaging before and after training and performed a voxel‐based morphometry (VBM) analysis to obtain information on the cerebellar and extracerebellar neural correlates of such training.

## 2 METHODS

### 2.1 Participants

A total of 41 patients with cerebellar degeneration and 44 neurologically healthy controls participated in the study. One patient and two controls dropped out prematurely because of acute illness unrelated to the study. Two controls had to be excluded from analysis because of incidental findings on brain magnetic resonance imaging (MRI). Hence, data from 40 patients (mean age 55 ± 11.4 years, 19 males) and 40 sex‐ and age‐matched controls (mean age 55.9 ± 10.9 years, 20 males) were included for analysis. Pretraining behavioral and MRI data of 30 patients and 30 controls of the present study population has been reported in a previous study by our group (Draganova et al., 2021).

All patients were diagnosed with a pure form of cerebellar cortical degeneration, primarily as spinocerebellar ataxia type 6 (SCA6), autosomal dominant cerebellar ataxia type 3 (ADCA III), and sporadic adult‐onset ataxia (SAOA) of unknown etiology. The severity of ataxia was assessed by the clinical Scale for the Assessment and Rating of Ataxia (SARA; Schmitz‐Hübsch et al., 2006). Patients and matched controls were pseudorandomly assigned to one of four training conditions (see below). The four subgroups of patients were matched for sex, age, and clinical ataxia rating (SARA) scores. Characteristics of individual patients and matched controls are detailed in Table 1. All participants were right‐handed as assessed by the Edinburgh‐handedness scale (Oldfield, 1971). The study was approved by the Ethics Committee of the Essen University Medical Center. Oral and written informed consent was obtained from all participants prior to testing.

**TABLE 1:** Clinical characteristics of cerebellar patients and matched controls. Patients and controls are grouped based on their assignments to the four training subgroups. Severity of ataxia was rated using the SARA (range SARA score 0–40; maximum SARA score = 40; Schmitz‐Hübsch et al., 2006). SCA 6, 8, 14 = spinocerebellar ataxia Types 6, 8, and 14; SAOA = sporadic adult‐onset ataxia; ADCA III = autosomal dominant cerebellar ataxia type III (pure cerebellar type); EOCA = early onset cerebellar ataxia. All patients suffered from cerebellar degeneration, and all patients presented with a pure cerebellar phenotype. Subject IDs refer to the order the patients and controls were recruited and assigned to the respective subgroups. For details on training conditions, see Table 2

	Cerebellar patients					Controls		
#	ID	Age (years)	Sex	Diagnosis	SARA score	ID	Age (years)	Sex
**Vision only**								
1	P04	56	F	SCA14	12	C05	59	F
2	P09	53	M	ADCAIII	26	C35	53	M
3	P12	53	F	SCA6	9	C30	57	F
4	P15	56	M	SCA6	14.5	C40	62	M
5	P20	65	F	ADCAIII	10.5	C04	68	F
6	P23	64	F	SAOA	13.5	C16	66	F
7	P26	62	M	SAOA	10.5	C42	62	M
8	P28	53	F	ADCAIII	9.5	C38	58	F
9	P39	49	M	SAOA	8	C15	50	M
10	P30	57	M	SCA8	9	C34	57	M
	**Mean *SD***	**57 5.29**	**5 F/5 M**	**Mean *SD***	**12.25 4.98**	**Mean *SD***	**59 5.51**	**5 F/5 M**
**Vision + Exp Feedb**								
1	P02	76	M	SAOA	12	C09	64	M
2	P06	53	F	SCA6	9.5	C24	53	F
3	P14	18	M	ADCAIII	4	C28	21	M
4	P16	60	F	SCA6	14	C23	65	F
5	P19	69	F	ADCAIII	20.5	C18	65	F
6	P25	37	M	SCA6	15.5	C07	37	M
7	P41	57	M	^a^Cerebellar degeneration	24	C21	67	M
8	P29	66	F	SAOA	8.5	C11	66	F
9	P37	49	M	ADCAIII	11	C39	53	M
10	P31	58	F	SCA8	8.5	C17	58	F
	**Mean *SD***	**54.30 16.75**	**5 F/5 M**	**Mean *SD***	**12.75 5.68**	**Mean *SD***	**54.90 15.07**	**5 F/5 M**
**No vision**								
1	P03	59	M	SCA6	7.5	C12	60	M
2	P07	51	F	ADCAIII	12.5	C01	51	F
3	P11	59	M	EOCA	22	C31	58	M
4	P13	53	M	ADCAIII	11	C44	46	M
5	P17	63	F	ADCAIII	13.5	C08	66	F
6	P18	50	F	ADCAIII	11.5	C43	55	M
7	P24	73	F	SAOA	10	C19	69	F
8	P32	33	M	EOCA	13	C10	34	M
9	P34	55	M	ADCAIII	9.5	C33	55	M
10	P36	44	F	EOCA	10	C29	44	F
	**Mean *SD***	**54.00 10.85**	**5 F/5 M**	**Mean *SD***	**12.05 3.73**	**Mean *SD***	**53.80 10.52**	**4 F/6 M**
**No vision + Exp Feedb**								
1	P01	66	M	SAOA	11	C02	67	M
2	P05	52	F	SCA14	12	C26	54	F
3	P08	69	F	SAOA	12.5	C03	71	F
4	P10	71	M	SAOA	15	C14	68	M
5	P21	47	M	SAOA	17.5	C37	47	M
6	P22	56	F	SAOA	14.0	C27	58	F
7	P27	37	M	EOCA	10	C22	34	M
8	P33	43	F	ADCAIII	6	C20	40	F
9	P40	50	F	SAOA	21	C32	57	F
10	P38	59	F	SCA6	3	C13	60	F
	**Mean *SD***	**55 11.33**	**6 F/4 M**	**Mean *SD***	**12.2 4.96**	**Mean *SD***	**55.60 12.18**	**6 F/4 M**

Abbreviation: SARA, Scale for the Assessment and Rating of Ataxia.

^a^ Post‐inflammatory.

### 2.2 Apparatus

Participants performed elbow flexion movements in the horizontal plane employing a one degree of freedom single‐joint manipulandum as described in Draganova et al. (2021).

**FIGURE 1:**
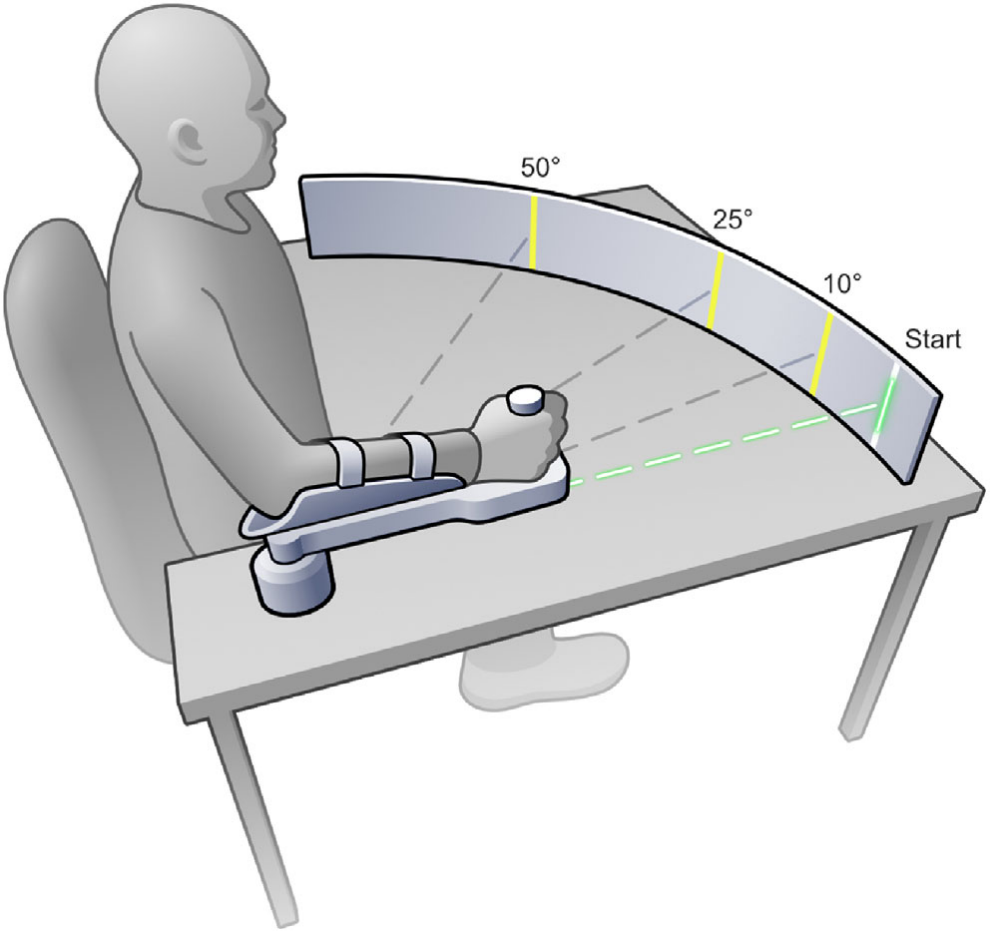
Experimental setup of the single‐joint manipulandum. Start position was at 90° elbow flexion. A low‐intensity laser attached to the manipulandum provided visual feedback of arm position online. An optical encoder embedded in the housing recorded forearm position

The manipulandum allowed the execution of precise goal directed movements without the need of the user to compensate for gravity (Figure 1). An optical encoder (US Digital H6, 2500 quadrature count/revolution; spatial resolution: 0.036°) housed under the rotating point of the lever arm recorded the angular position of forearm at a sampling rate of 102.5 Hz. Participants sat in front of the manipulandum and placed their arm on the manipulandum lever. Chair height and lever handle placement were adjusted to the anthropometrics of each participant such that the joint axis of the elbow and the encoder shaft axis aligned.

The start position of the forearm was at 90° elbow flexion (neutral position; defined as 0° by the encoder). Targets were strips attached to a metal semicircular frame at a distance of 95 cm from the axis of rotation of the manipulandum. After a “start” command given by the experimenter, participants moved the forearm to the target in a single, ramp‐like movement without subsequent correction of position error. Participants held their arm in their end position for 4 s and then moved the arm back to the start position. Participants were instructed to perform swift (but not as fast as possible) and accurate movements. During testing, the experimenter controlled the speed and gave additional instruction to correct it, if the speed was too slow or too high according to previous calculated limits.

### 2.3 Training procedure

Using the single‐joint manipulandum, participants performed goal‐directed elbow flexion movements with their right arm. Patients and matched controls were pseudorandomly assigned to one of four training conditions (*N* = 10 per subgroup): Condition 1 consisted of conventional visuomotor training without additional explicit movement error feedback (**Vision Only**). Condition 2 added additional explicit feedback to the same visuomotor training as condition 1 (**Vision + Exp Feedb**). Condition 3 occluded vision. Only proprioceptive online feedback was available to the trainee (**No Vision**), and condition 4 consisted of the same proprioceptive training as condition 3 but participants received additional explicit error feedback (**No Vision + Exp Feedb**) (Table 2).

**TABLE 2:** Summary of the four training groups. Feedback types were different for each training condition. Online proprioceptive feedback was available during motion, either with or without explicit feedback. During the conditions with vision, learning was driven by visual and proprioceptive inputs. No vision conditions constituted forms of proprioceptive training. Information about the *movement goal* (i.e., whether target has been reached or not) was available either through vision (condition 1, 2), or through explicit verbal feedback (condition 3, 4). Verbal feedback about the *magnitude and direction of the movement error* was provided after movement execution only in the “**+ Exp Feedb”** conditions (e.g., “Target was undershoot by xx degrees. Increase movement by x degrees.”)

Condition	Training group	Online sensory feedback		Explicit verbal error feedback	Movement goal feedback
		Visual	Proprioceptive		
1	**Vision**	Yes	Yes	No	Visual
2	**Vision + Exp Feedb**	Yes	Yes	Yes	Visual
3	**No Vision**	No	Yes	No	Verbal
4	**No Vision + Exp Feedb**	No	Yes	Yes	Verbal

For the training conditions with **Vision**, during pointing, participants received visual forearm position feedback from a green laser pointer attached to the distal end of the manipulandum. For the **No Vision** (proprioceptive) training conditions, participants wore a mask that fully occluded vision. At the beginning of each trial, the investigator manually guided the arm of the participant from the start position to the target. Participants memorized the target position of his/her elbow before going back to the start position. Thereafter, participants actively moved their arm from the start position to the memorized target position in a single movement without any correction holding the arm at the end position for about 4 s. Then, the investigator provided verbal feedback whether the target was reached yes or no. Thereafter the investigator manually corrected the arm position by moving the forearm to the middle of the target zone (proprioceptive feedback). The manual correction served to indicate the spatial discrepancy between the actual and desired position. After experiencing the correct target position for 4 s, the participant moved his arm back to the start position and the next trial was started.

For the **Exp Feedb** training conditions, participants received explicit verbal information (a) about their final joint position (JP) at the end of the reach in relation to the target, and (b) by providing explicit instructions on how to minimize future movement errors (i.e., “Target was undershoot by xx degrees. Increase movement by x degrees” or “Target was overshoot by xx degrees. Decrease movement by x degrees”).

All participants received information about the movement goal (i.e., the target) either visually, and/or as verbal feedback (i.e., “on target,” “target has not been reached”) when vision was blocked. Online proprioceptive feedback was always available.

#### 2.3.1 Training sequence

Training was performed on five consecutive days. Targets were at 10 and 50° of elbow flexion. Training consisted of four consecutive blocks per day. Each block comprised 25 trials of one of the target amplitudes (10 or 50°) (total trials per day = 100). Blocks of target amplitudes with 10 and 50° alternated. Amplitude of the first block was counterbalanced between participants in each subgroup: 50% of the participants started with Amplitude 10° on Day 1, and 50% with Amplitude 50°. The amplitude in the first block alternated between training days. On each day, training started with three familiarization trials to the target amplitude of the first block (with vision in the **Vision** conditions, and without vision in the **No Vision** conditions).

There were 3 min rest periods after each block of training. After completing two blocks, participants took their arm out of the manipulandum to relax the arm. In the explicit feedback conditions (**+Exp Feedb**), a scale was attached to the metal semicircular target frame indicating the distance from the target in degrees. The scale was also shown during familiarization trials in those conditions with explicit feedback. To assess fatigue, movement velocity was measured online, and additional pauses were made as needed.

In all conditions, the width of the target strips attached to the semicircular frame was adjusted to the level of performance achieved by the participant. Five different levels were determined: Level 5 = target width 4.5 cm (2.7° of arc), Level 4 = 3.5 cm (2.11°), Level 3 = 2.5 cm (1.5°), Level 2 = 1.5 cm (0.9°), and Level 1 with target width = 0.5 cm (0.3°). Participants started at the maximum target width (Level 5) and only after five successful consecutive movements, the target width was changed to the next more difficult level. A movement was considered successful, if the beam of the laser pointer fell into the target zone. Participants were informed about the five levels prior to the experiment. During the experiment, they received oral feedback when they had reached the next level. If the participant achieved Level 1, the target zone remained the same until the end of training.

Duration for a single trial was set to 10 s. With feedback and pause, each trial lasted about 15–20 s in the **Vision** training conditions, and about 50–60 s in the **No Vision** training conditions. Training lasted 45–60 min on each day in the **Vision** training conditions, and 90–100 min in the **No Vision** training conditions.

### 2.4 Assessment of training

On the days before and after training, motor performance was measured similar to the **Vision Only** condition. That is, vision was available, but no explicit forms of movement feedback were given, to allow direct comparison of motor performance outcomes between the four training conditions. Targets had a width of 0.3°, and were at 10, 25, and 50° of elbow flexion. Participants performed 10 trials per target amplitude (30 trials total). Targets were presented in pseudorandom order with maximal two consecutive movements of the same target amplitude. The order of targets was the same across all participants.

#### 2.4.1 Measurements

The calculation of the behavioral parameters is described in detail in Draganova et al. (2021) and is reproduced below. Data from the optical encoder were processed offline by custom written software in MATLAB (MathWorks, Natick, MA). For each trial the absolute *JP error* (JPE) and the *relative JPE* (RJPE) with respect to each target amplitude was computed. RJPE was calculated based on the instantaneous JP and JPE over a period of 408 samples, which corresponded to the 4 s holding period of the arm (i.e., after the transport phase of the movement was completed):

$$\mathrm{JPE}=\left(\sum_{j=1}^{n}\mathrm{JP}\left(j\right)/n\right)-\mathrm{TA}$$

$$\text{RJPE}=\left|\;\frac{\mathrm{JPE}}{\mathrm{TA}}\right|$$

where *N* = 408 is the number of sampling points covering 4 s holding period when the target was reached, TA is the corresponding amplitude 10, 25, or 50°. RJPE was expressed in percentage of the amplitude (i.e., RJPE was multiplied by 100). In addition, the peak velocity (*V*_max_) during the transport phase of the pointing movement was determined.

All movement trials were visually inspected for data integrity prior to inclusion in the analysis. In the pretraining and post‐training assessments, one trial each had to be excluded in four controls and three patients because of technical errors. In the training sessions, no trials had to be excluded. For each participant and target amplitude, means (*M*) and *SD*s of RJPE were calculated on each day (pre, post, and the five training days).

#### 2.4.2 Statistical analysis

The primary outcome parameter was the difference in mean RJPE (RJPE M for each of the movement amplitudes before and after training. Secondary outcome parameter was the *SD* of RJPE (RJPE *SD*) calculated for each of the movement amplitudes. In addition, training‐related changes of RJPE M and RJPE *SD* were assessed across the 5 days of training.

The data for RJPE M and RJPE *SD* were not normally distributed. Therefore, they were modeled and analyzed using the nonparametric rank‐based analysis of variance (ANOVA)‐type test for factorial longitudinal data using the statistical software packages nparLD (http://www.R-project.org/) and SAS (Domhof & Langer, 2002; Noguchi, Gel, Brunner, & Konietschke, 2012). The underlying treatment effects are so‐called relative effects, also known as Wilcoxon–Mann–Whitney effects *p*_*X*_ = *P*(*X* < *Y*), where *X* denotes the factor level of interest and *Y* denotes the fixed reference (mean) distribution. The effects display the order of the data across all groups: If *p*_*X*_ < *p*_*Z*_, then the data under condition X tends to be smaller than those measured under condition Z. If *p*_*X*_ = *p*_*Z*_, then none of the data under conditions X and Z tend to be smaller or larger. The nonparametric rank‐based method allows reliable conclusions when sample sizes are small. Since the procedure is solely based on ranks of the data, presence of outliers do not affect the outcome.

Considering RJPE M and RJPE *SD* as dependent variables, two independent ***Group*s** (cerebellar patients vs. healthy controls), four independent ***Training conditions*** (**Vision Only** vs. **Vision + Exp Feedb** vs. **No Vision** vs. **No Vision + Exp Feedb**), the repeated factor ***Time*** (pre vs. post; the five training days, respectively), and their interactions were included in the model. Statistics were calculated separately for each of the amplitudes (10, 25, and 50° pre/post; 10 and 50° training). Mean *V*_max_ (across the 10 trials per movement amplitude in pre/post, and across the 50 trials per amplitude and day in training) was introduced in the statistical model as covariate of no interest to correct for possible differences in movement velocity, across days and between patients and controls. The significance level was set to *p* < .05, whereas all results are interpreted in an exploratory and not in a confirmatory manner.

### 2.5 Acquisition and analysis of MRI data

MRI acquisition and analysis procedures are described in detail in Draganova et al. (2021). On the days before and after training, structural MRI scans were acquired from all participants using a 3T human whole body combined MRI‐PET system (Siemens Healthcare, Erlangen, Germany) and a 16‐channel head/neck‐array coil (Siemens Healthcare). Whole‐brain T1‐weighted magnetization prepared rapid acquisition gradient echo (MP‐RAGE) images were acquired (isotropic voxel size of 1 mm; TE = 3.26 ms; TR = 2,530 ms; inversion time = 1,100 ms; FA = 7°; acquisition matrix = 256 × 256 × 176; BW = 200 Hz/Px; GRAPPA with *R* = 2 and 48 reference lines; TA = 6:24 min:s).

#### 2.5.1 Voxel‐based morphometry

VBM analysis was performed considering the whole brain using the MNI normalization procedure (Ashburner & Friston, 2000). We refrained from doing separate normalization procedures of the cerebellum and cerebrum, because it has been shown by Abdelgabar et al. (2019) that SUIT normalization, which has been developed specifically to normalize the cerebellum, is not superior compared to current MNI normalization procedures considering the cerebellum (which agrees with our experiences).

#### 2.5.2 Standard whole‐brain VBM preprocessing for longitudinal data

The T1‐weighted MRI scans were preprocessed using the default longitudinal approach implemented in the CAT12 toolbox (http://dbm.neuro.uni-jena.de/cat/, release 1447) incorporated in the Statistical Parametric Mapping software package ‐ SPM12 (Welcome Department of Cognitive Neurology, London, UK, http://www.fil.ion.ucl.ac.uk/spm). The post‐training scans were registered to the baseline (pretraining) scans for each participant separately. A mean of the realigned images was generated for each participant and used for bias correction for field inhomogeneity between the different time points. Based on the segmentation (tissue classification) of the mean image, using tissue probability maps, tissue was classified into gray matter (GM), white matter (WM), and cerebrospinal fluid (CSF). Using two transformations, linear (12 parameters affine) and nonlinear transformations (warping), the mean image was registered to match a standard template (DARTEL) within a unified model (Ashburner & Friston, 2005). The spatial normalization parameters estimated during this step resulted in spatial deformation fields. The latter were applied to the GM segmentations of the images of both time points (pretraining and post‐training). To correct the volume changes after spatial normalization, GM density segments were modulated by the Jacobian determinants as derived from the spatial normalization's deformation parameters (Kurth, Thompson, & Luders, 2018). Quality control was achieved based on visual inspection of individual raw and preprocessed MRI scans for artifacts. None of the scans revealed artifacts. Furthermore, the check‐data‐quality toolbox in CAT12 was employed to quantitatively assess image and preprocessing quality. Weighted overall quality measure (IQR) was good (pretraining: controls: mean 86.21%, *SD* 0.41, range: 84.38–86.57; patients: mean 86.05%, *SD* 0.50, range 84.08–86.51; post‐training: controls: mean: 84.17%, *SD* 0.53, 86.55–86.04, patients: mean: 85.22%, *SD* 0.33, range 86.55–86.24). In addition, visual inspection of the normalized individual MRI scans ensured that normalization process resulted in biologically plausible results. Finally, the modulated normalized GM segments were smoothed with a Gaussian kernel of 8 mm full width at half‐maximum. The individual GM, WM, CSF volumes and total intracranial volume (TIV) were estimated in CAT12 by the TIV function.

Given the small sample size of each training subgroup (*N* = 10), and because the amount of learning considering our main outcome parameter (RJPE M) did not differ between groups and training conditions (***Group*** × ***Training Condition*** × ***Time*** interaction effects >0.05; see Section 3), decision was made to collapse the data and perform VBM analysis based on the group of all cerebellar patients (*N* = 40) and all controls (*N* = 40) independent of the training condition. First, and most importantly, we were interested in GM tissue volume changes before and after training. The statistical analysis was performed by setting up a flexible factorial design in the linear general model framework. The main effects of ***Time*** (pre < post) were calculated for cerebellar patients and controls by paired‐samples *t* test. F contrasts were calculated to assess ***Group*** (patients vs. controls) by ***Time*** interactions. TIV and age were not considered in the (repeated measures) flexible factorial design because they are practically constant in that case. That is, TIV is unlikely to change within a week and an age difference of 7 days (before and after training) is negligible (http://www.neuro.uni-jena.de/cat12/CAT12-Manual.pdf). It is known that constant covariates have no effect on repeated measures factors (see, e.g., https://imaging.mrc-cbu.cam.ac.uk/statswiki/FAQ/constantcov; Van Breukelen & Van Dijk, 2007), and are usually not considered in flexible factorial designs (Bezzola, Mérillat, Gaser, & Jäncke, 2011; Burciu et al., 2013; Taubert et al., 2010).

Whole brain analysis was performed and results are reported using an exploratory, uncorrected threshold of *p* = .001. To partially correct for multiple comparisons the expected voxels per cluster (<*k*>) calculated based on random field theory in SPM12 were used as a cluster size threshold (cf. Burciu et al., 2013). Assignment of peak MNI coordinates and brain clusters to brain areas was done using the *Julich*‐Brain Cytoarchitectonic *Atlas* (JuBrain), based on the maximum probability map (MPM) (Eickhoff et al., 2007). In case, the respective brain area was not part of the current JuBrain atlas the automated anatomical labeling (AAL3) atlas was used instead (Rolls, Huang, Lin, Feng, & Joliot, 2020; Tzourio‐Mazoyer et al., 2002).

Next, significant brain cluster regions considering the main effects of ***Time*** (pre < post) in patients and controls, and considering the F‐contrast (interaction ***Group*** by ***Time***) were used as volumes of interest (VOIs). All masks were generated using the xjView toolbox (https://www.alivelearn.net/xjview). They were resliced to achieve the voxel size of the segmented modulated images. Individual GM volumes (GMVs) were calculated for each VOI by multiplying the VOI mask with the segmented modulated individual images using the get_totals MATLAB function (http://www0.cs.ucl.ac.uk/staff/g.ridgway/vbm/get_totals.m). As data were normally distributed, repeated measures ANOVA were performed with individual GMV in a given VOI as dependent variable, group (cerebellar vs. control) as between subject factor and time (pre vs. post) as within subject factor using SPSS software (IBM Corp. Released 2019. IBM SPSS Statistics for Windows, Version 26.0. Armonk, NY: IBM Corp.). Greenhouse–Geyser correction was applied where appropriate.

Finally, we were interested in differences in GM tissue volume between patients and controls prior to training. A two‐sample *t* test was performed comparing the baseline (pretraining) GMV between cerebellar patients and controls including age and TIV as covariates of no interest. Multiple regression analyses were performed comparing baseline GMV with RJPE M and SARA score in the group of all cerebellar patients. Age and TIV were included as covariates of no interest.

## 3 RESULTS

### 3.1 Training‐related changes in motor performance

#### 3.1.1 Pre‐ versus post‐training

**FIGURE 2:**
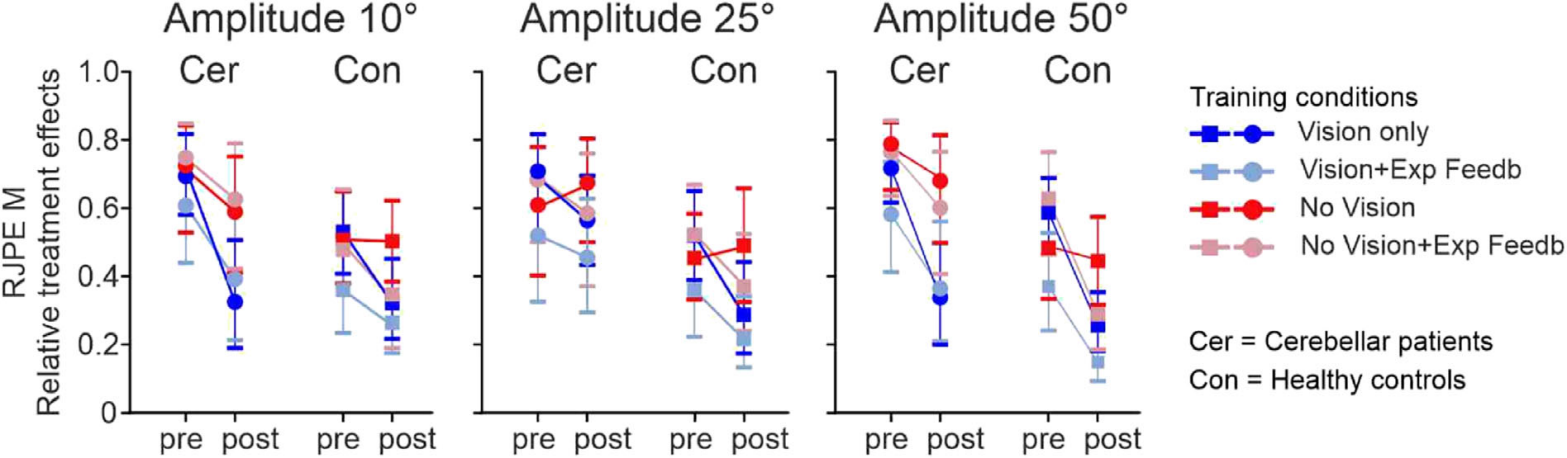
Relative treatment effects comparing mean *relative joint position error* (RJPE M) between pretraining and post‐training for movement amplitudes 10° (a), 25° (b), and 50° (c) in cerebellar patients (Cer) and controls (Con). Median relative treatment effects and 95% confidence intervals are shown comparing the four training conditions (dark blue: Vision Only, light blue: Vision + Exp Feedb; dark red: No Vision, light red: No Vision + Exp Feedb) in cerebellar patients (indicated by circles) and control participants (indicated by squares)

**FIGURE 3:**
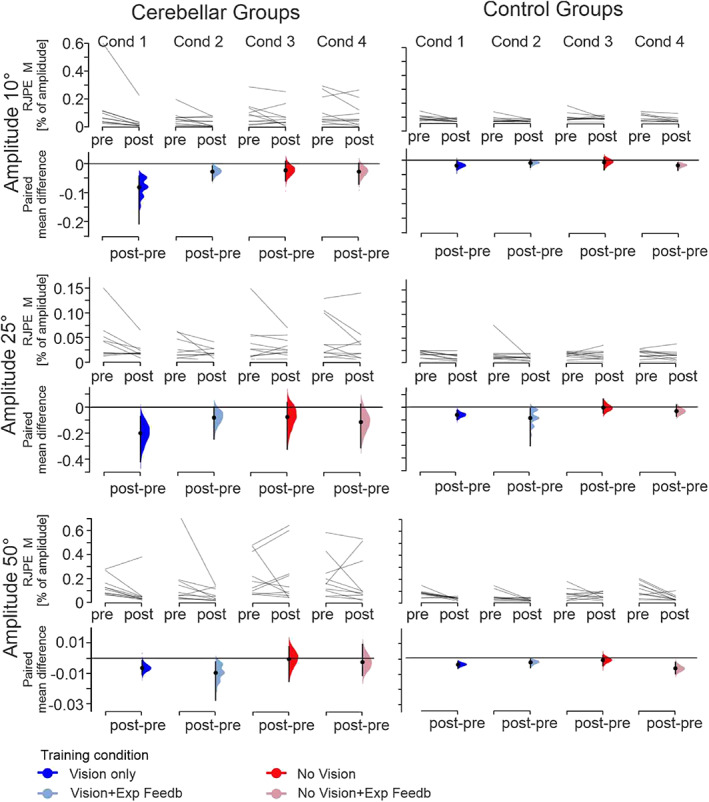
Multipaired estimation plots displaying individual data points and effect sizes comparing mean *relative joint position error* (RJPE M) between the pretraining and post‐training (post) for movement amplitudes 10, 25, and 50° in cerebellar patients and controls. Thin lines in upper panels represent individual data points (pre vs. post) of each participant. Lower panels show effects sizes. Black dots represent mean differences between pretraining and post‐training assessment in each subgroup and error bars 95% confidence intervals (CI). 95% CI are calculated by bootstrap resampling (Ho, Tumkaya, Aryal, Choi, & Claridge‐Chang, 2019). Filled curves represent the bootstrap sampling distribution of the observed data. Multipaired estimation plots were generated using the web‐application of DABEST (“data analysis with bootstrap‐coupled estimation”; http://www.estimationstats.com/)

The main finding was that RJPE M was smaller at post‐training when compared to pretraining in each of the four training conditions in cerebellar patients, and that the training effects were not less compared to controls. Training effects were most prominent in the two movement amplitudes (10 and 50°) which were trained. Relative treatment effects are shown in Figure 2, and individual data in Figure 3.

Nonparametric statistics revealed a significant effect of ***Time*** considering each of the three movement amplitudes (all *p* values <.05). A significant ***Training condition*** × ***Time*** interaction effect was found for movement amplitude 50° (*p* < .05), but not for movement amplitude 25 and 10° (*p* values >.05). Cerebellar patients and controls showed less training‐related reduction of RJPE M in the training conditions without vision compared to the training conditions with vision with the exception of the training condition 4 (No Vision + Exp Feedb) in controls. A ***Training condition*** effect was found for movement amplitude 50° (*p* < .05), but not for movement amplitude 25 and 10° (*p* values >.05). Although great care was taken to match cerebellar patients in the four training subgroups according to SARA score (Table 1), baseline RJPE performance was different between training conditions in cerebellar patients, but also controls (Figure 3) explaining the ***Training condition*** effect. ***Group*** effects were significant (*p* values <.05) confirming that controls had a better visuomotor performance than patients. ***Group*** × ***Training condition***, ***Group*** × ***Time***, or ***Group*** × ***Training condition*** × ***Time*** interaction effects were not observed (*p* values >.05). As noted above, mean *V*_max_ was introduced in the statistical model as covariate of no interest. The effect of velocity (mean *V*_max_) was significant considering amplitude 10° (*p* < .05), but not for the amplitudes 25 and 50° (*p* > .05). Statistical results are summarized in Table 3.

**TABLE 3:** Summary of statistical results considering pretraining and post‐training assessments. Nonparametric rank‐based ANOVA‐type tests for factorial longitudinal data were applied. Degrees of freedom were adjusted in case variances differed

Amplitude	Effect	RJPE M	RJPE *SD*
10°	Group	*F*(1,59.9) = 12.59; *p* = .0008	*F*(1,72) = 15.53; *p* = .0002
	Training condition	*F*(2.87, 59.9) = 2.06; *p* = .1181	*F*(3,72) = 3.54; *p* = .0212
	Group × training condition	*F*(2.87, 59.7) = 0.71; *p* = .5439	*F*(3,72) = 0.21; *p* = .8791
	Time (pre vs. post)	*F*(1,68.6) = 20.43; *p* < .0001	*F*(1,71) = 23.46; *p* < .0001
	Group × time	*F*(1,58.9) = 2.02; *p* = .1601	*F*(1,71) = 0.16; *p* = .6920
	Training condition × time	*F*(2.85, 59.3) = 2.36; *p* = .0835	*F*(3,71) = 2.29; *p* = .0882
	Group × condition × time	*F*(2.85, 58.9) = 0.88; *p* = .4521	*F*(3,71) = 0.95; *p* = .4206
	Velocity/covariate of no interest	*F*(1,87.3) = 7.98; *p* = .0059	*F*(1,71) = 9.43; *p* = .0029
25°	Group	*F*(1,61.3) = 15.66; *p* = .0002	*F*(1,72) = 16.88; *p* = .0001
	Training condition	*F*(2.92, 61.6) = 1.92; *p* = .1371	*F*(3,72) = 2.47; *p* = .0702
	Group × training condition	*F*(2.92, 61.5) = 0.04; *p* = .9889	*F*(3,72) = 0.08; *p* = .9706
	Time (pre vs. post)	*F*(1,75.7) = 8.35; *p* = .0050	*F*(1,71) = 15.43; *p* = .0002
	Group × time	*F*(1,67.8) = 2.04; *p* = .1576	*F*(1,71) = 0.03; *p* = .8647
	Training condition × time	*F*(2.98, 68.7) = 1.54; *p* = .2128	*F*(3,71) = 4.18; *p* = .0104
	Group × condition × time	*F*(2.97, 67.9) = 0.09; *p* = .9668	*F*(3,71) = 0.51; *p* = .6696
	Velocity/covariate of no interest	*F*(1,117) = 0.63; *p* = .4276	*F*(1,71) = 9.47; *p* = .0030
50°	Group	*F*(1,52.7) = 23.23, *p* < .0001	*F*(1,72) = 22.05; *p* < .0001
	Training condition	*F*(2.78, 52.7) = 5.84, *p* = .0021	*F*(3,72) = 4.54; *p* = .0066
	Group × training condition	*F*(2.77, 52.6) = 0.62, *p* = .5949	*F*(3,72) = 0.41; *p* = .7395
	Time (pre vs. post)	*F*(1,63.7) = 51.94, *p* < .0001	*F*(1,71) = 46.89; *p* < .0001
	Group × time	*F*(1,62.3) = 0.17, *p* = .6821	*F*(1,71) = 0.20; *p* = .6569
	Training condition × time	*F*(2.91, 62.3) = 4.02, *p* = .0119	*F*(3,71) = 3.74; *p* = .0173
	Group × condition × time	*F*(2.91, 62.1) = 0.73, *p* = .5318	*F*(3,71) = 0.65; *p* = .5783
	Velocity/covariate of no interest	*F*(1,59.3) = 2.15, *p* = .1479	*F*(1,71) = 0.02; *p* = .8783

Abbreviations: ANOVA, analysis of variance; RJPE, *relative joint position error*.

Comparable training effects were found for movement variability (RJPE *SD*). Nonparametric analysis revealed a significant effect of ***Time*** with smaller RJPE *SD* in post‐training compared to pretraining considering the three movement amplitudes (all *p* values <.05). The ***Training condition*** × ***Time*** interaction was significant for movement amplitude 25° (*p* < .05) and 50° (*p* < .05). Both cerebellar patients and controls showed less training‐related reduction of RJPE *SD* in the training conditions without vision compared to the training conditions with vision (Figures 1, 2 and 1, 3 Supplementary Materials). The ***Group*** effects were significant (*p* values <.05). ***Group*** × ***Time***, ***Group*** × ***Training condition***, and ***Group*** × ***Training Condition*** × ***Time*** did not interact (*p* values >.05). A main effect for ***Training condition*** was found for 10 and 50° (*p* values <.05). The effect of velocity (mean *V*_max_; covariate of no interest) was significant considering amplitudes 10 and 25° (*p* values <.05), but not 50° (*p* > .05). Statistical results are summarized in Table 3.

#### 3.1.2 Performance change across the five training days

**FIGURE 4:**
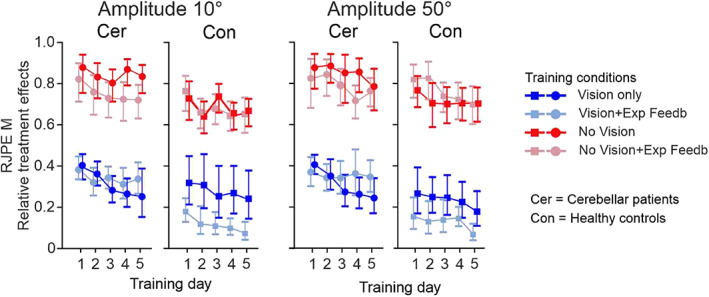
Relative treatment effects for mean *relative joint position error* (RJPE M) across the five training days considering movement amplitudes 10 and 50° in cerebellar patients (Cer) and controls (Con). Median relative treatment effects and 95% confidence intervals are shown comparing the four training conditions

Both patients with cerebellar degeneration and healthy controls improved RJPE M across the five training days in both the 10 and the 50° movement amplitude conditions. As expected overall performance was worse in the two training conditions without vision compared to the two training conditions with vision. Relative treatment effects are shown in Figure 4.

Nonparametric statistics revealed a significant effect of ***Time*** (training Days 1–5), ***Training condition*** (1–4), and ***Group*** (*patients* vs. *controls)* for both amplitudes (*p* values <.05). No significant interactions were observed (*p* values >.05). The velocity (mean *V*_max_) covariate does not seem to impact the response (*p* > .05). Statistical results are summarized in Table 4. Group mean data are given in Table 1, 4 in Supplementary Materials.

**TABLE 4:** Summary of statistical results considering the five training days. Nonparametric rank‐based ANOVA‐type tests for factorial longitudinal data were applied. Degrees of freedom were adjusted in case variances differed

Amplitude	Effect	RJPE M	RJPE *SD*
10°	Group	*F*(1,50.9) = 18.69; *p* < .0001	*F*(1,56.7) = 17.35; *p* = .0001
	Training condition	*F*(2.7, 50.9) = 96.70; *p* < .0001	*F*(2.79, 56.3) = 94.08; *p* < .0001
	Group × training condition	*F*(2.7, 50.9) = 2.34; *p* = .0906	*F*(2.79, 56) = 2.12; *p* = .1116
	Time (training Days 1–5)	*F*(3,155) = 15.16; *p* < .0001	*F*(3.17, 141) = 10.96; *p* < .0001
	Group × time	*F*(3.02, 153) = 0.81; *p* = .4925	*F*(3.2, 139) = 1.37; *p* = .2524
	Training condition × time	*F*(7.64, 153) = 1.30; *p* = .2517	*F*(8.15, 139) = 1.02; *p* = .4220
	Group × condition × time	*F*(7.64, 153) = 1.21; *p* = .2968	*F*(8.15, 139) = 0.86; *p* = .5568
	Velocity/covariate of no interest	*F*(1,31.3) = 0.01; *p* = .9337	1*F*(1,35.8) = 151.54; *p* < .0001
50°	Group	*F*(1,61.2) = 20.38; *p* < .0001	*F*(1,63.7) = 21.54; *p* < .0001
	Training condition	*F*(2.91, 61) = 119.11; *p* < .0001	*F*(2.95, 63.6) =109.98; *p* < .0001
	Group × training condition	*F*(2.91, 61) = 2.37; *p* = .0812	*F*(2.95, 63.5) =3.12; *p* = .0328
	Time (training Days 1–5)	*F*(3.17, 164) = 11.41; *p* < .0001	*F*(3.34, 132) = 10.90; *p* < .0001
	Group × time	*F*(3.18, 163) = 0.30; *p* = .8331	*F*(3.34, 131) = 0.88; *p* = .4613
	Training condition × time	*F*(8.45, 163) = 1.31; *p* = .2395	*F*(8.19, 131) = 1.44; *p* = .1845
	Group × condition × time	*F*(8.45, 163) = 0.98; *p* = .4531	*F*(8.19, 131) = 1.27; *p* = .2623
	Velocity/covariate of no interest	*F*(1,78.4) = 0.25; *p* = .6178	*F*(1,117) = 94.63; *p* < .0001

Abbreviations: ANOVA, analysis of variance; RJPE, *relative joint position error*.

Comparable training effects were observed considering movement variability (Figure 1, 4 and Table 2, 4 in Supplementary materials). RJPE *SD* showed significant effects of ***Time*** (training Days 1–5), ***Training condition*** and ***Group*** (patients vs. controls) for both amplitudes (*p* values <.05). Interaction effects were not significant (*p* values <.05), except a significant ***Group*** × ***Training condition*** effect (*p* < .05) for movement amplitude 50° that was due to the reduction of movement variability in the proprioceptive training conditions in cerebellar patients when compared to controls. The effect of velocity (mean *V*_max_; covariate of no interest) was significant considering both amplitudes (*p* values <.05). Statistical results are summarized in Table 4.

#### 3.1.3 Movement velocity

Mean *V*_max_ was not significantly different between the cerebellar patient and control group (all *p* values >.05, nonparametric statistics). Both cerebellar patients and controls showed a significant decline of velocity comparing the pretraining and post‐training assessments, and across the five training days (all *p* values <.05, see Supplementary Materials for group mean data (Tables 1, 3 and 3, 4) and summary of statistics (Tables 2, 3 and 4‐4). However, the reported training effects cannot be explained by a speed‐accuracy trade‐off because mean *V*_max_ had been introduced in the statistical model as covariate of no interest.

### 3.2 Training‐related changes in GMV

Given the small sample size of each training subgroup (*N* = 10), and because the amount of learning considering our main outcome parameter (RJPE M) did not differ between groups and training conditions (***Group*** × ***Training Condition*** × ***Time*** interaction effects >0.05) the decision was made to perform VBM analysis comparing all cerebellar patients, and all control subjects independent of the training condition. Furthermore, as outlined in methods, the decision was made to perform an exploratory analysis considering the whole brain and using an uncorrected threshold of *p* < .001 (partially corrected for multiple comparisons using predetermined cluster size).

**FIGURE 5:**
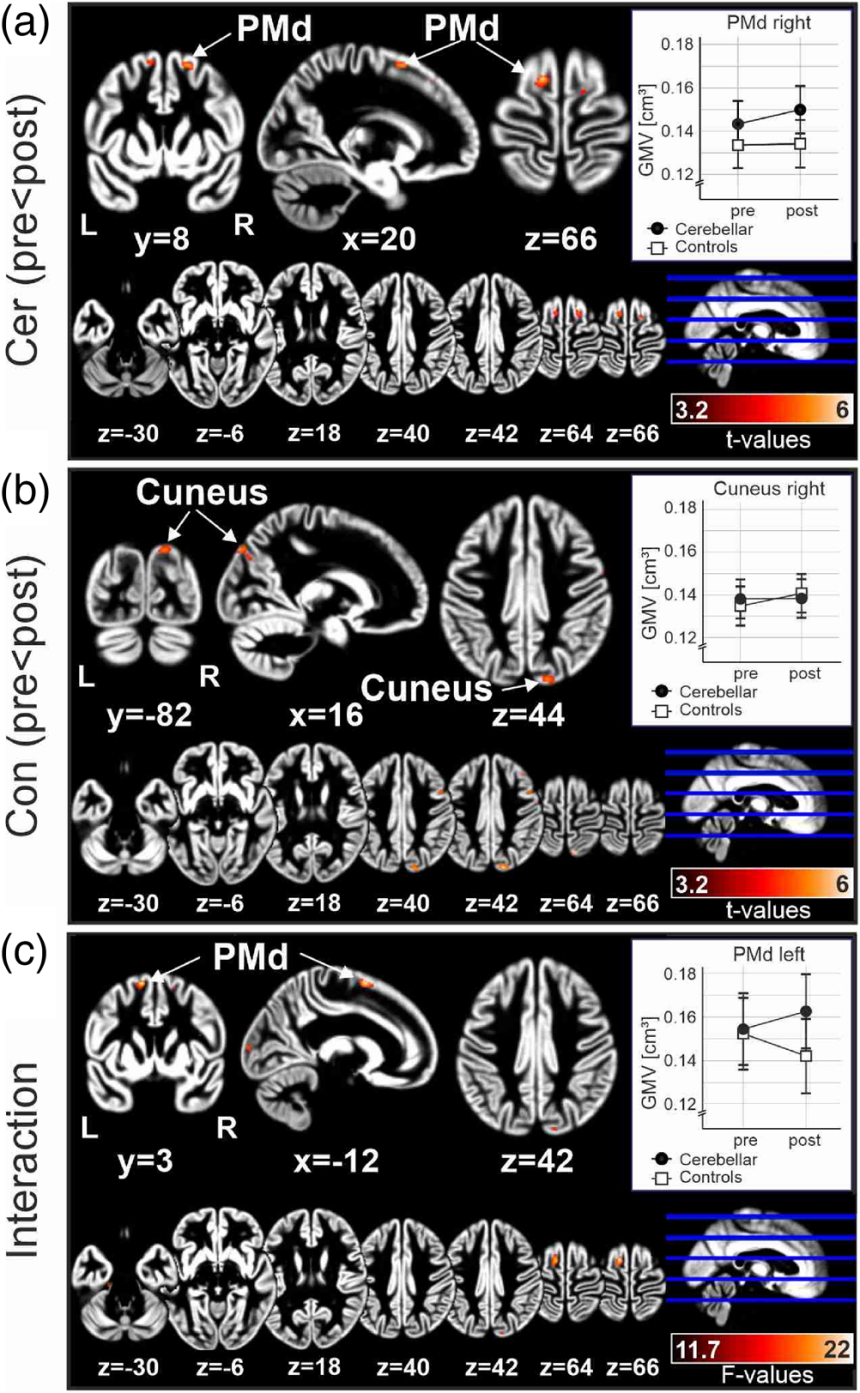
Training‐related increases of gray matter volume (GMV) in patients with cerebellar degeneration (Cer) and healthy controls (Con). (a) t‐Contrast pretraining < post‐training in the group of all cerebellar patients, (b) t contrast pretraining < post‐training in the group of all healthy controls. (c) F contrast of the interaction time (pretraining < post‐training) and group (cerebellar vs. controls). Voxel‐based morphometry (VBM) data are shown at an exploratory threshold of *p* < .001 overlaid on the mean smoothed gray matter (GM) segmentation image in all cerebellar patients (a), all controls (b), and all patients and controls (c). In each panel, VBM clusters are shown superimposed on coronal, sagittal and axial sections (upper part), as well as axial sections (lower part), the latter being the same in (a–c) for direct comparison. Small inserts show mean GMV and *SE*s in the largest cluster of the given contrast assessed in individual cerebellar patients and controls pretraining and post‐training

A flexible factorial analysis revealed training‐related increases of GMV in patients with cerebellar degeneration and healthy controls. The brain areas involved, however, were different between patients and controls (Figure 5). In the group of all patients with cerebellar degeneration, GMV increased primarily in the dorsolateral premotor cortex (PMd; area 6d2, 6d3, Sigl, 2018; t‐contrast pre < post; threshold *p* < .001, extent threshold *k*_*e*_ > 30.96; Figure 5a). Increase was present on both sides, with a larger cluster on the right compared to the left. On the left, there was a small extension into the supplementary motor area (SMA; 6mr/preSMA; Ruan et al., 2018). In addition, GMV increased in the primary sensory cortex on the right (Area 2; Grefkes, Geyer, Schormann, Roland, & Zilles, 2001) with a small extension into area 7PC of the superior parietal lobe (SPL) (Scheperjans et al., 2008) (not shown).

In controls, on the other hand, GM increase was observed primarily within the right cuneus (including areas hPO1 and hIP8 in the posterior intraparietal sulcus with some extension to area 7P in the superior partial cortex (SPL), Richter et al., 2019; t‐contrast pre < post; threshold *p* < .001, extent threshold *k*_*e*_ > 30.96; Figure 5b). Smaller clusters of GM increases were also found in the right middle frontal gyrus (Figure 5b) and left Crus I (not shown).

The ***Group*** (patients vs. controls) by ***Time*** interaction is shown in Figure 5c. At the threshold of *p* < .001 and corrected extended voxel threshold of 25.2 a significant interaction was found in left dorsolateral premotor cortex (Area 6d2; Sigl, 2018) with a small extension into the SMA (6mR/preSMA; Ruan et al., 2018).

Findings are summarized in Table 5. For further illustration, GMV was calculated in individual patients and controls in three VOIs. The largest cluster of GM increase in the three main contrasts outlined above were considered which were within the right PMd (Figure 5a), the right cuneus (Figure 5b) and the left PMd (Figure 5c). Mean GMV in these VOIs in the pretraining and post‐training assessments in patients and controls are shown in the small, white inserts in Figure 5a–c. GMV increase in PMd was present in cerebellar patients, but not in controls. Within the cuneus, GMV increase was present in controls, but not in patients, and it appeared to be overall smaller than the GMV increase in PMd in the patients. Repeated measures ANOVA was performed with GMV as dependent variable for each of the three VOIs. Within subject factor was ***Time*** (pre vs. post) and between subject factor ***Group*** (cerebellar vs. control). Considering the VOI in right PMd, ANOVA revealed a significant ***Time*** effect (*F*(1,78) = 9.38, *p* = .003) and a significant ***Time*** by ***Group*** interaction (*F*(1,78) = 6.36, *p* = .014). The ***Group*** effect was not significant (*p* = .09). Considering the VOI in right cuneus, the ***Time*** effect (*F*(1,78) = 6.28, *p* = .014) and the ***Group*** by ***Time*** interaction effects (*F*(1,78) = 5.42, *p* = .023) were significant. The ***Group*** effect was not significant (*p* = .94). Considering the VOI in the left PMd, there was no significant effect of ***Time*** (*p* = .72), but a significant ***Time*** × ***Group*** interaction effect (*F*(1,78) = 9.89, *p* = .023). The ***Group*** effect was not significant (*p* = .33).

**TABLE 5:** Voxel‐based morphometry comparing GMV before and after training

AAL atlas	JuBrain atlas MPM			MNI peak coordinate (mm)				
Peak voxel assignment	Peak voxel assignment	Cluster assignment	Side	*x*	*y*	*z*	*k*_*E*_ */*<*k*>	
**Cerebellar patients (pre < post)** *t*‐value								
Frontal_Sup^a^	Assigned to Area 6d2^b^ 38.6%^c^—Area 6d2 3.9%—Area 6d3	65.1%^d^ in Area 6d2 13.6% in Area 6d3	R	18	9	63	107/**<30.96>**	4.90
Supp_Motor_Area	Assigned to Area 6d2 62.4%—Area 6d2 21.1%—Area 6d1		R	15	0	69		
Supp_Motor_Area	Assigned to Area 6d2 70.4%—6d2	86.2% in Area 6d2 13.8% in Area 6mr/preSMA	L	−11	8	66	44	4.23
Post_central	Assigned to Area 2 64.3%—Area 2 17.5%—Area 7PC 15.3%—Area hIP3 2.2%—Area 3b	92.1% in Area 2 7.5% in Area 7PC (SPL)	R	29	−41	56	67	4.07
**Controls (pre < post)** *t*‐value								
Cuneus	25.5%—Area hPO1 10.2%—Area hIP8 9.2%—Area 7P	38.6% in Area hPO1 (IPS) 14.3% in Area 7P (SPL) 3% in Area hIP8 (IPS)	R	17	−83	42	92/**<30.96>**	4.58
Cuneus	18%—Area hPO1		R	15	−77	36		
Frontal_Mid			R	42	20	50	68	3.91
Cerebellum_Crus1			L	−47	−57	−36	38	3.76
**Interaction time (pre < post) × group (cerebellar vs. control)** *F*‐value								
Supp_Motor_Area	Assigned to Area 6d2 65.5%—Area 6d2	95.9% in Area 6d2 2.7% in Area 6mr/preSMA	L	−12	6	66	120/**<25.20>**	21.02

*Note*: Results of whole brain analysis reported at an exploratory, uncorrected threshold of *p* = .001, partially corrected for multiple comparisons using predetermined cluster sizes (<*k*>, expected voxels per cluster). *k*_*E*_ = voxels per cluster; MNI coordinate = Montreal Neurological Institute coordinates.

Abbreviations: GMV, gray matter volume; MPM, maximum probability map; SPL, superior parietal lobe.

^a^ AAL3 atlas labels (Rolls et al., 2020): Frontal_Sup = superior frontal gyrus, dorsolateral; Supp_Motor_Area = supplementary motor area; Post_central = postcentral gyrus; Cuneus = cuneus; Frontal_Mid = middle frontal gyrus; Cerebellum_Crus1 = Crus I of cerebellar hemisphere.

^b^ JuBrain atlas labels (Eickhoff et al., 2007): area 6d2, area 6d3 = dorsolateral premotor areas; 6mr/preSMA = supplementary motor area; area 2 = primary sensory cortex; areas 7PC, 7P = areas the SPL; areas hPO1, hIP8 = areas in the posterior intraparietal sulcus.

^c^ Probabilities for all histological data found at the position of this voxel (Eickhoff et al., 2007; see also https://www.fz‐juelich.de/SharedDocs/Downloads/INM/INM7/EN/SPM_Toolbox/Manual.pdf?__blob=publicationFile).

^d^ Relative extent (i.e., percentage) of cluster assigned to a cytoarchitectonic area based on the cytoarchitectonic MPM (Eickhoff et al., 2007).

**FIGURE 6:**
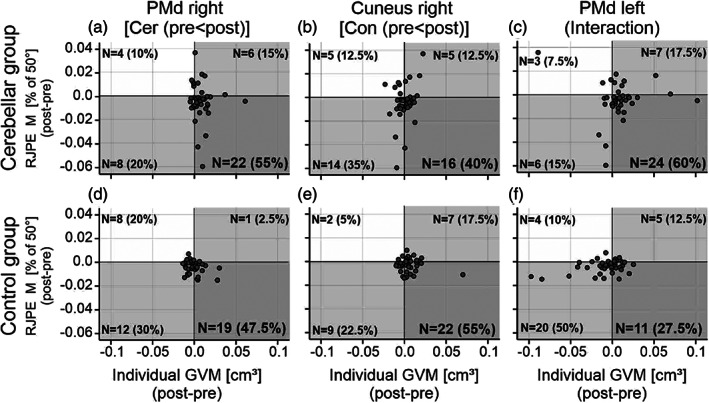
Comparison of training‐related changes (post‐training minus pretraining) in gray matter and in motor performance (mean *relative joint position error* (RJPE M) for amplitude 50°). Each data point represents an individual participant. Panels (a–c) show the data of individual cerebellar patients, (d–f) for controls. Gray matter change is shown in three volumes of interests (VOIs): dorsolateral premotor cortex (PMd) on the right and left, and cuneus. Data are based on findings of the flexible factorial analysis shown in Figure 5. Dots localized to the right of the vertical zero line represent participants revealing a training‐related increase of gray matter volumes (GMVs). Dots localized below the horizontal zero line represent participants revealing training‐related improvement of movement performance (i.e., decrease of RJPE M). Thus, *N* (%) in the dark gray quadrants represent the number of participants (% of the group) who showed gray matter increase in a given VOI and improved motor performance

To analyze whether GMV increases were correlated with improved motor performance, we asked the question how many cerebellar patients and how many controls showed both an increase of GMV and improved RJPE M in the three VOIs. Increase in GMV was defined as a difference of GMV in a given VOI that was larger at post‐training when compared to pretraining. No threshold was used. As shown in Figure 6a,d, we found that 55% (*N* = 22 out of a total of *N* = 40) of the cerebellar patients, and 47.5% (19/40) of the controls, showed both an increase of GMV in right PMd as well as a decrease of RJPE M (in the 50° movement amplitude condition). Within the right cuneus (Figure 6b,e), 55% (22/40) of the controls, and 40% (16/40) of the patients showed an increase of GMV and a decrease of RJPE M, whereas 60% (24/40) of the patients, and 27.5% (11/40) of the controls showed an increase of GMV and a decrease of RJPE M within the left PMd (**Figure** **6**c,f). Thus, in the left PMd area, which showed a significant ***Group*** by ***Time*** interaction effect, the majority of patients and the least controls, showed both an increase of GMV volume and an improvement of motor performance. Correlation analyses, however, did not reveal significant correlations between change in GMV in the three VOIs and change in RJPE M (Kendall's tau‐b: 0.06–0.175; all *p* values >.05) except for GMV (post‐pre) in right cuneus which was positively correlated with RJPE M (post‐pre) in cerebellar patients (Kendall's tau‐b: 0.183, *p* = .048).

### 3.3 Differences in GMV between cerebellar patients and controls at baseline

**FIGURE 7:**
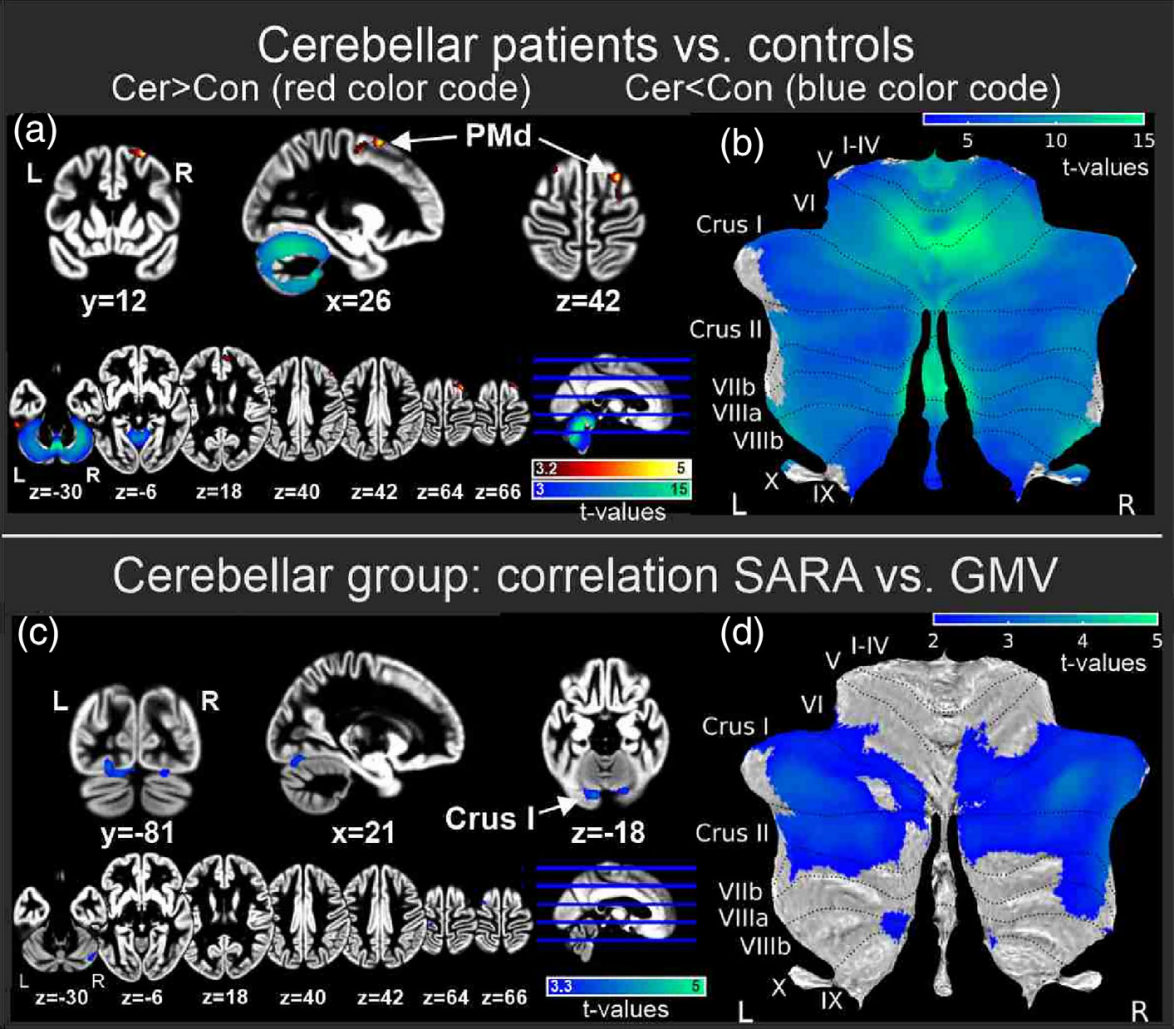
Gray matter volumes (GMVs) at baseline (pretraining). (a,b) Comparing all patients with cerebellar degeneration and all healthy control participants. The t contrast cerebellar (Cer) patients < controls (Con) is shown in blue/green colors and the t contrast Con > Cer in red/yellow colors. Significant differences are shown (a) superimposed on coronal, sagittal, and axial sections of the whole brain map (calculated as mean of cerebellar and control group gray matter [GM] images), and (b) superimposed on a flat map of the cerebellum (Diedrichsen & Zotow, 2015) at a threshold of *p* < .05, FWE corrected. (c,d) Multiple regression analysis showing significant positive correlations between total SARA score and gray matter values in cerebellar patients (*p* < .001; extent threshold: *k*_*e*_ > 79.4). I‐X indicate cerebellar lobules based on Schmahmann et al. (1999)

Finally, we were interested if GMV differences existed in cerebellar patients and controls already at baseline. As expected, patients with cerebellar degeneration exhibited smaller GMV in most parts the cerebellar cortex (Figure 7a,b). Differences were most marked in anterior cerebellar lobe and adjacent lobule VI, as well as in the vermis (threshold: *p*_FWE_ < .05; see also Table 5; Diedrichsen, Balsters, Flavell, Cussans, & Ramnani, 2009). Furthermore, the decrease of cerebellar GMV in lobules Crus I and II as well as VIIIB correlated with an increase of ataxia severity based on the SARA score (*p* < .001, extent threshold correction: *k*_*e*_ > 79.4); Figure 7c,d). Using the same threshold, no significant negative correlations between GMV were found when comparing with RJPE M and cerebellar GMV. No significant decreases were found in the cerebral cortex. Cerebellar patients showed increased GMV compared to controls in the dorsal premotor cortex (PMd; Area 6d2; Sigl, 2018), Broca area (Areas 45, OP8, OP9; Amunts et al., 2010), anterior cingulate (Area p23; Palomero‐Gallagher et al., 2019), and midfusiform gyrus (Area FG4; Lorenz et al., 2017) at a threshold of *p* < .001 (partially corrected for multiple comparisons using an extent threshold of *k*_*e*_ > 94.76; see also Table 6).

**TABLE 6:** Voxel‐based morphometry comparing gray matter volume (GMV) between cerebellar patients and control participants at baseline (pretraining)

AAL atlas	JuBrain atlas^a^ MPM			MNI peak coordinate (mm)				
Peak voxel/cluster assignment	Peak voxel assignment	Cluster assignment	Side	*x*	*y*	*z*	*k*_*E*_ */*<*k*>	*t*‐Value
**Cerebellar patients < controls**								*p*_FWE_ = .05
**Total cluster: Vermis_6** ^b^				**5**	**−63**	**−24**	**35,176**	**17.32**
Cerebellum_6			R				3,387	
Cerebellum_6			L				3,300	
Cerebellum_Crus1			L				2,952	
Cerebellum_8			R				2,676	
Cerebellum_8			L				2,137	
Cerebellum_4_5			L				1,952	
Cerebellum_Crus2			L				1,766	
Cerebellum_Crus2			R				1,672	
Cerebellum_4_5			R				1,669	
Cerebellum_9			R				1,150	
Cerebellum_9			L				794	
Vermis_4_5							733	
Vermis_6							597	
Vermis_8							521	
Cerebellum_7b			L				512	
Cerebellum_7b			R				436	
Vermis_7							368	
Vermis_9							282	
Vermis_3							248	
Cerebellum_10			R				102	
Cerebellum_3			R				96	
Vermis_10							44	
Vermis_1_2							31	
Cerebellum_10			L				20	
**Cerebellar patients > controls**								*p* < .001
Frontal_Sup^c^	13.6%^e^—Area 6d2^d^	29%^f^ in Area 6d3 4.2% in Area 6d2	R	27	14	65	149 **<94.76>**	5.06
Frontal_Inf_Tri	Assigned to Area 45 36.2%—Area 45 23.6%—Area OP9 9.6%—OP8 8%—Area 44	32.8% in Area 45 26.4% in Area 44 25.7% in Area OP9 7.3% in Area OP8	L	−54	24	0	185	4.50
Frontal_Mid			R	47	33	35	262	4.39
			R	44	32	42		
Frontal_Sup_Medial		65.1% in Area p32	L	3	53	24	533	4.24
	47.4%—Area p32		R	12	51	17		
Temporal_Inf		4.8% in Area FG4	L	−53	−38	−30	152	4.09
Frontal_Inf_Tri	Assigned to Area 45 65.3%—Area 45 17.9%—Area 44	79.6% in Area 45 19.8% in Area 44	L	−57	20	24	93	3.82
Frontal_Mid			L	−21	30	57	111	3.73

*Note*: Cerebellar patients < controls: results of whole brain analysis corrected at a corrected threshold of *p* < .05, FWE corrected; cerebellar patients > controls results of whole brain analysis reported at an exploratory, uncorrected threshold of *p* = .001, partially corrected for multiple comparisons using predetermined cluster sizes (<*k*>, expected voxels per cluster). *k*_*E*_ = voxels per cluster; MNI coordinate = Montreal Neurological Institute coordinates.

Abbreviation: MPM, maximum probability map.

^a^ Note that the current version of the JuBrain atlas includes the cerebellar nuclei, but not the cerebellar cortex.

^b^ AAL atlas labels of cerebellar lobules identified by anatomical locator of the xjview SPM toolbox (https://www.alivelearn.net/xjview).

^c^ AAL3 atlas labels (Rolls et al., 2020): Frontal_Sup = superior frontal gyrus, dorsolateral; Frontal_Inf_Tri = inferior frontal gyrus, triangular part; Frontal_Mid = middle frontal gyrus; Frontal_Sup_Medial = superior frontal gyrus, medial; Temporal_Inf = inferior temporal gyrus; cerebellum_ = lobules of the cerebellar hemispheres; Vermis_ = lobules of the cerebellar vermis.

^d^ JuBrain atlas labels (Eickhoff et al., 2007): Area 6d2, Area 6d3 = dorsolateral premotor areas; Areas 44, 45, OP9 = Broca area; Area p23 = anterior cingulate: Area FG4 = midfusiform gyrus.

^e^ Probabilities for all histological data found at the position of this voxel (Eickhoff et al., 2007; see also https://www.fz‐juelich.de/SharedDocs/Downloads/INM/INM7/EN/SPM_Toolbox/Manual.pdf?__blob=publicationFile).

^f^ Relative extent (i.e., percentage) of cluster assigned to a cytoarchitectonic area based on the cytoarchitectonic MPM (Eickhoff et al., 2007).

## 4 DISCUSSION

This study investigated to what extent upper limb motor learning is preserved in cerebellar degeneration. We systematically investigated, if the provision of explicit verbal feedback could “boost” the learning outcome for cerebellar patients. In addition, we examined if such motor learning was driven primarily by visual feedback, or if these patients were still able to use proprioceptive information as an error feedback signal. The three main findings of the study are the following: First, explicit verbal feedback did not enhance visuomotor learning in the cerebellar patient group. Second, in our sample of patients, who presented with mild to severe degenerative ataxia, proprioceptive‐based motor learning was preserved. Third, as a neural correlate of motor learning the control group exhibited an increase in GMV (GMV) most prominently in visual association cortices, while motor learning in the cerebellar patients was associated with a GMV increase in premotor cortex. Results corroborate previous findings of our group in a balance training task in patients with cerebellar degeneration (Burciu et al., 2013), and suggest that compensatory remodeling primarily takes place in those cerebral motor areas that receive strong efferent projections from the cerebellum.

### 4.1 Explicit verbal feedback does not aid motor learning in cerebellar disease

Different to our expectation, additional explicit verbal feedback about movement errors and instruction on how to control for them, did not lead to superior learning neither in patients with cerebellar degeneration nor in controls. This finding is at odds with earlier reports showing that cerebellar patients can use explicit information to apply cognitive strategies during visuomotor adaptation to minimize movement error (Taylor et al., 2010; Wong, Marvel, Taylor, & Krakauer, 2019). In one experiment (Taylor et al., 2010), cerebellar patients received incongruent visual feedback (cursor and physical hand position were shifted by 45°) and successfully applied a −45° strategy to compensate for the visual error. That is, they were able to use a cognitive strategy. In our case, participants did not have to adapt to an external perturbation. They received explicit verbal feedback about the magnitude and the direction of the movement error to optimize motor outcome in a skill (i.e., pointing as precisely as possible). This explicit feedback augmented existing visual and proprioceptive feedback about the arm position. The fact that neither controls nor cerebellar patients benefitted from this feedback suggests that this feedback was redundant, as it did not enhance performance. It also implies that cerebellar patients cannot easily substitute possible deficits in visual (Maschke, Gomez, Tuite, Pickett, & Konczak, 2006) or proprioceptive perception (Bhanpuri et al., 2012) through the use of explicit verbal error feedback to improve motor performance. It is plausible that the implicit, cerebellar‐dependent components of learning prevailed (Kim, Ogawa, Lv, Schweighofer, & Imamizu, 2015; McDougle, Bond, & Taylor, 2015; Smith, Ghazizadeh, & Shadmehr, 2006).

### 4.2 Cerebellar patients can use proprioceptive error feedback for motor learning

We sought to elucidate, if the known deficits in motor learning of cerebellar patients can be explained by an inability to use proprioceptive information when learning a goal‐directed behavior. We here show that patients with degenerative ataxia are still capable to use proprioceptive information about a limb position to improve the spatial precision of goal‐directed pointing movement. As expected, spatial precision to reach an external target was lower when vision was occluded, and participants had to rely solely on proprioceptive position signals. Furthermore, the amount of learning tended to be smaller in the training conditions without vision compared to conditions with vision particularly for the learning‐related decrease of variability. However, there were no differences between groups. This is encouraging from a physical rehabilitation perspective as it indicates that motor learning through the use of proprioceptive signals is at least partially preserved in patients with cerebellar degeneration. Thus, proprioceptive training may be a useful addendum to conventional training with visual feedback in patients with cerebellar degeneration.

### 4.3 Training‐related GM increases in premotor cortex

The most important finding of the present study was that training effects in cerebellar patients were related to GM increases primarily within the premotor cortex. This result is in good accordance with a previous study of our group, which also found training‐related GM increases in the premotor cortex in a postural training task (Burciu et al., 2013). Premotor cortex is involved in the generation of motor plans based on visuospatial information from the parietal cortex, and in motor learning (Hardwick et al., 2015; Mazurek & Schieber, 2017). Both the present arm movement task and the previous balance task involved movements to visual targets, in the latter by moving the center of gravity on a force platform. Knowing that premotor cortex receives large efferent projections from the cerebellum (Bostan, Dum, & Strick, 2013), a GM increase in premotor cortex may be an attempt of the cortical targets of cerebellar output to compensate for the altered cerebellar signals. This implies that GMV increase in premotor cortex constitutes a neurostructural response to altered efferent input. From a computational motor control perspective, it may be viewed as an attempt by the system to maintain network integrity. Given that cerebellar output signals modulate motor planning, a GMV increase associated with cerebellar degeneration could be understood as an effort to add neural resources to interpret increasingly noisier cerebellar efferent signals and to use such signals for motor planning and learning (e.g., as a predictive or error feedback signal). Predictive ability is not limited to the cerebellum (Sokolov, Miall, & Ivry, 2017) and may also be a function of premotor cortex (Stadler et al., 2012). Premotor cortex has also been reported to compensate for M1 lesions due to stroke for upper limb (for review, see Kantak, Stinear, Buch, & Cohen, 2012) and gait function (Miyai et al., 2002). Thus, premotor cortex appears to be an important hub in compensatory remodeling following damage of the motor system.

The present findings may be relevant for clinical practice. For example, premotor cortex may be a possible target for noninvasive brain stimulation (NIBS) to enhance the effects of motor training in cerebellar patients. So far, studies using NIBS focused on stimulating the cerebellum, M1, or the spinal cord (Benussi et al., 2017; Benussi et al., 2018; Hulst et al., 2017).

In addition to premotor cortex, we saw learning‐related GM increase in the SMA. The SMA is part of the basal ganglia circuit involved in motor learning, and there is recent evidence that the cerebellum has direct anatomical connections not only with PM, but also with SMA (Bostan et al., 2013). In good agreement with the present study, dynamic causal modeling analysis of fMRI data showed that the SMA was involved in motor learning in patients with degenerative cerebellar disease (Tzvi et al., 2017). One may assume that those cerebral areas, which are involved in learning mechanisms which are less cerebellar‐dependent primarily contribute to the compensation of learning deficits such as prefrontal cortex (related to explicit strategic learning; Taylor & Ivry, 2014), basal ganglia (related to reward‐based learning; Schultz, 2006) and primary motor cortex (related to use‐dependent learning; Orban de Xivry, Criscimagna‐Hemminger, & Shadmehr, 2011). This, however, was not the case. Very similar to our previous study (Burciu et al., 2013), parts of the cerebello‐cortical‐motor‐loop unaffected by the disease showed the most significant learning‐related GM increase.

The pattern of learning‐related GM increases was very different in controls, where most learning‐related changes were found in visual associative areas. The same area has been found to show changes in regional brain morphology related to learning of a more complex visuomotor task, that is juggling, in healthy participants by Scholz, Klein, Behrens, and Johansen‐Berg (2009) and Draganski et al. (2004). Similar to the present findings, increased GMV was not observed in motor cortical areas (M1, premotor cortex, SMA) for reasons incompletely understood (Draganski et al., 2004; Scholz et al., 2009).

Finally, similar to our previous study, cerebellar GMV increase related to motor learning was absent in cerebellar patients and scant in controls. This does not exclude that learning‐related plastic changes take place within the cerebellum in cerebellar patients, which has been shown in histological data of training studies in mouse models of cerebellar degeneration (e.g., Fucà et al., 2017).

### 4.4 Limitations

The main limitation of our study is weak statistical power. Although the total number of patients (*N* = 40) was comparatively large, there were only 10 participants per training group given the between‐subject design. We cannot exclude that differences between training conditions become obvious in larger patient populations. Furthermore, imaging data analysis was only partially corrected for multiple comparisons. However, because our main finding of training‐related GMV increase in cerebellar patients' premotor cortex are supported by previous data of our group (Burciu et al., 2013), we believe that our current findings are valid.

Distribution of diagnoses differed between training subgroups (Table 1). However, great care was taken to enroll only patients with a pure form of cerebellar cortical degeneration. Thus, although the etiology differed between patients, the underlying pathology was the same. Furthermore, we assured that there was significant overlap between groups with all groups including patients with SCA6, ADCAIII, and SAOA. Therefore, it is unlikely that the comparatively small differences in distribution of diagnoses between subgroups had a significant impact on our results.

Another limitation is training duration. Five days is a comparatively short training duration. Effects of the training conditions may become more pronounced with longer training duration. Finally, elbow flexion is a relatively simple, single‐joint movement. Because arm ataxia becomes more pronounced in multijoint movements (Bastian, Martin, Keating, & Thach, 1996), findings need to be validated in future studies using more complex movements.

## 5 CONCLUSIONS

Our data confirm that patients with cerebellar degeneration still benefit from motor training. We found no evidence that providing additional explicit verbal feedback effectively “boosts” sensorimotor learning. Consequently, there is still a need to further understand under which conditions cerebellar patients may benefit from explicit movement instructions. In contrast, the same patients effectively used proprioceptive information for motor learning when vision was blocked. Most importantly, our data provide additional evidence that premotor areas are involved in compensatory processes in cerebellar disease. Future studies are needed to understand to which extent premotor cortex can functionally compensate for cerebellar dysfunction.

## CONFLICT OF INTEREST

The authors declare no potential conflict of interests.

## Supporting information


**Figure S1** Relative treatment effects comparing RJPE SD between pre‐training and post‐training for movement amplitudes 10° (A), 25° (B) and 50° (C) in cerebellar patients (Cer) and controls (Con). Median relative treatment effects and 95% confidence intervals are shown comparing the four training conditions (dark blue: Vision Only, light blue: Vision + Exp Feedb; dark red: No Vision, light red: No Vision + Exp Feedb) in cerebellar patients (indicated by circles) and control participants (indicated by squares).
**Figure S2** Multi‐paired estimation plots displaying individual data points and effect sizes comparing RJPE SD between the pre‐ and post‐training (post) for movement amplitudes 10°, 25° and 50° in cerebellar patients and controls. Thin lines in upper panels represent individual data points (pre vs. post) of each participant. Lower panels show effects sizes. Black dots represent mean differences between pre‐ and post‐training assessment in each subgroup and error bars 95% confidence intervals (CI). 95% CI are calculated by bootstrap resampling (Ho et al., 2019). Filled curves represent the bootstrap sampling distribution of the observed data. Multi‐paired estimation plots were generated using the web‐application of DABEST (“data analysis with bootstrap‐coupled estimation”; http://www.estimationstats.com/).
**Figure S3** Relative treatment effects for RJPE SD across the five training days considering movement amplitudes 10° and 50° in cerebellar patients (Cer) and controls (Con). Median relative treatment effects and 95% confidence intervals are shown comparing the four training conditions
**Table S1** Mean peak velocity (V_max_) – pre‐training/post‐training. V_max_ is expressed in degree per second (°/s). Group means and standard deviations comparing pre‐training (Pre) and post‐training (Post) assessments in **A)** cerebellar patients and **B)** controls of the three movement amplitudes (10°, 25°, 50°) in the four training conditions (cond): 1 = **Vision Only;** 2 = **Vision + Exp Feedb;** 3 = **No Vision;** 4 = **No Vision + Exp Feedb**
**Table S2** Mean peak velocity (V_max_) – pre‐training vs. post‐training. Summary of statistical results considering pre‐training and post‐training assessments. Nonparametric rank‐based ANOVA‐type tests for factorial longitudinal data were applied. Degrees of freedom were adjusted in case variances differed.
**Table S3** RJPE mean (RJPE M) – five training days. RJPE M is expressed as percentage of the movement amplitude. Group means and standard deviations across the five training days in **A)** cerebellar patients and **B)** controls of the two movement amplitudes (10°, 50°) in the four training conditions (cond): 1 = **Vision Only;** 2 = **Vision + Exp Feedb;** 3 = **No Vision;** 4 = **No Vision + Exp Feedb**
**Table S4** RJPE standard deviation (RJPE SD) – five training days. RJPE SD is expressed as percentage of the movement amplitude. Group means and standard deviations across the five training days in **A)** cerebellar patients and **B)** controls of the two movement amplitudes (10°, 50°) in the four training conditions (cond): 1 = **Vision Only;** 2 = **Vision + Exp Feedb;** 3 = **No Vision;** 4 = **No Vision + Exp Feedb**
**Table S5** Mean peak velocity (V_max_) – five training days. V_max_ is expressed in degree per second (°/s). Group means and standard deviations across the five training days in **A)** cerebellar patients and **B)** controls of the two movement amplitudes (10°, 50°) in the four training conditions (cond): 1 = **Vision Only;** 2 = **Vision + Exp Feedb;** 3 = **No Vision;** 4 = **No Vision + Exp Feedb**
**Table S6** Mean peak velocity (V_max_) – five training days. Summary of statistical results considering the five training days. Nonparametric rank‐based ANOVA‐type tests for factorial longitudinal data were applied. Degrees of freedom were adjusted in case variances differed.

## Data Availability

The data that support the findings of this study are available on request from the corresponding author. The data are not publicly available due to privacy or ethical restrictions.
