# Supplementary material for: Motor training‐related brain reorganization in patients with cerebellar degeneration
Source: Hum Brain Mapp. 2021 Dec 11;43(5):1611–29. doi: 10.1002/hbm.25746 (PMC8886660; doi:10.1002/hbm.25746)
Supplement: Supplementary file 1 — Figure S1 Relative treatment effects comparing RJPE SD between pre‐training and post‐training for movement amplitudes 10° (A), 25° (B) and 50° (C) in cerebellar patients (Cer) and controls (Con). Median relative treatment effects and 95% confidence intervals are shown comparing the four training conditions (dark blue: Vision Only, light blue: Vision + Exp Feedb; dark red: No Vision, light red: No Vision + Exp Feedb) in cerebellar patients (indicated by circles) and control participants (indicated by squares). Figure S2 Multi‐paired estimation plots displaying individual data points and effect sizes comparing RJPE SD between the pre‐ and post‐training (post) for movement amplitudes 10°, 25° and 50° in cerebellar patients and controls. Thin lines in upper panels represent individual data points (pre vs. post) of each participant. Lower panels show effects sizes. Black dots represent mean differences between pre‐ and post‐training assessment in each subgroup and error bars 95% confidence intervals (CI). 95% CI are calculated by bootstrap resampling (Ho et al., 2019). Filled curves represent the bootstrap sampling distribution of the observed data. Multi‐paired estimation plots were generated using the web‐application of DABEST (“data analysis with bootstrap‐coupled estimation”; http://www.estimationstats.com/). Figure S3 Relative treatment effects for RJPE SD across the five training days considering movement amplitudes 10° and 50° in cerebellar patients (Cer) and controls (Con). Median relative treatment effects and 95% confidence intervals are shown comparing the four training conditions Table S1 Mean peak velocity (Vmax) – pre‐training/post‐training. Vmax is expressed in degree per second (°/s). Group means and standard deviations comparing pre‐training (Pre) and post‐training (Post) assessments in A) cerebellar patients and B) controls of the three movement amplitudes (10°, 25°, 50°) in the four training conditions (cond): 1 = Vision Only; 2 = Vision + Exp Feedb; 3 [file HBM-43-1611-s001.docx]

**Supplementary Figures**

**
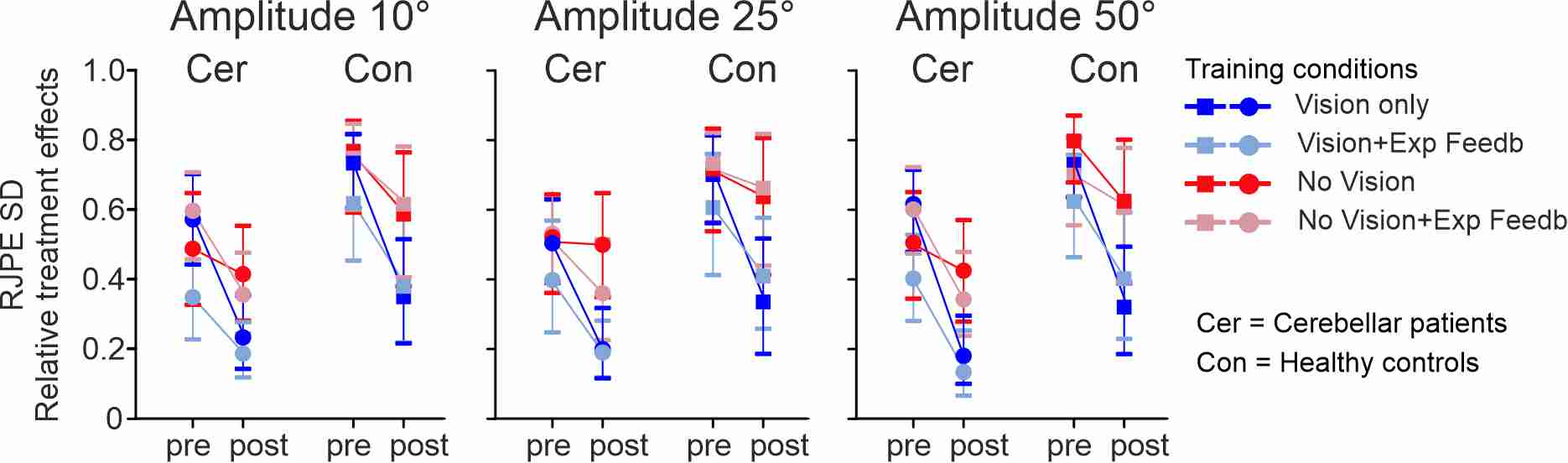
**

**Fig. 2-1**

Relative treatment effects comparing RJPE SD between pre-training and post-training for movement amplitudes 10° (**A**), 25° (**B**) and 50° (**C**) in cerebellar patients (Cer) and controls (Con). Median relative treatment effects and 95% confidence intervals are shown comparing the four training conditions (dark blue: Vision Only, light blue: Vision + Exp Feedb; dark red: No Vision, light red: No Vision + Exp Feedb) in cerebellar patients (indicated by circles) and control participants (indicated by squares).

**
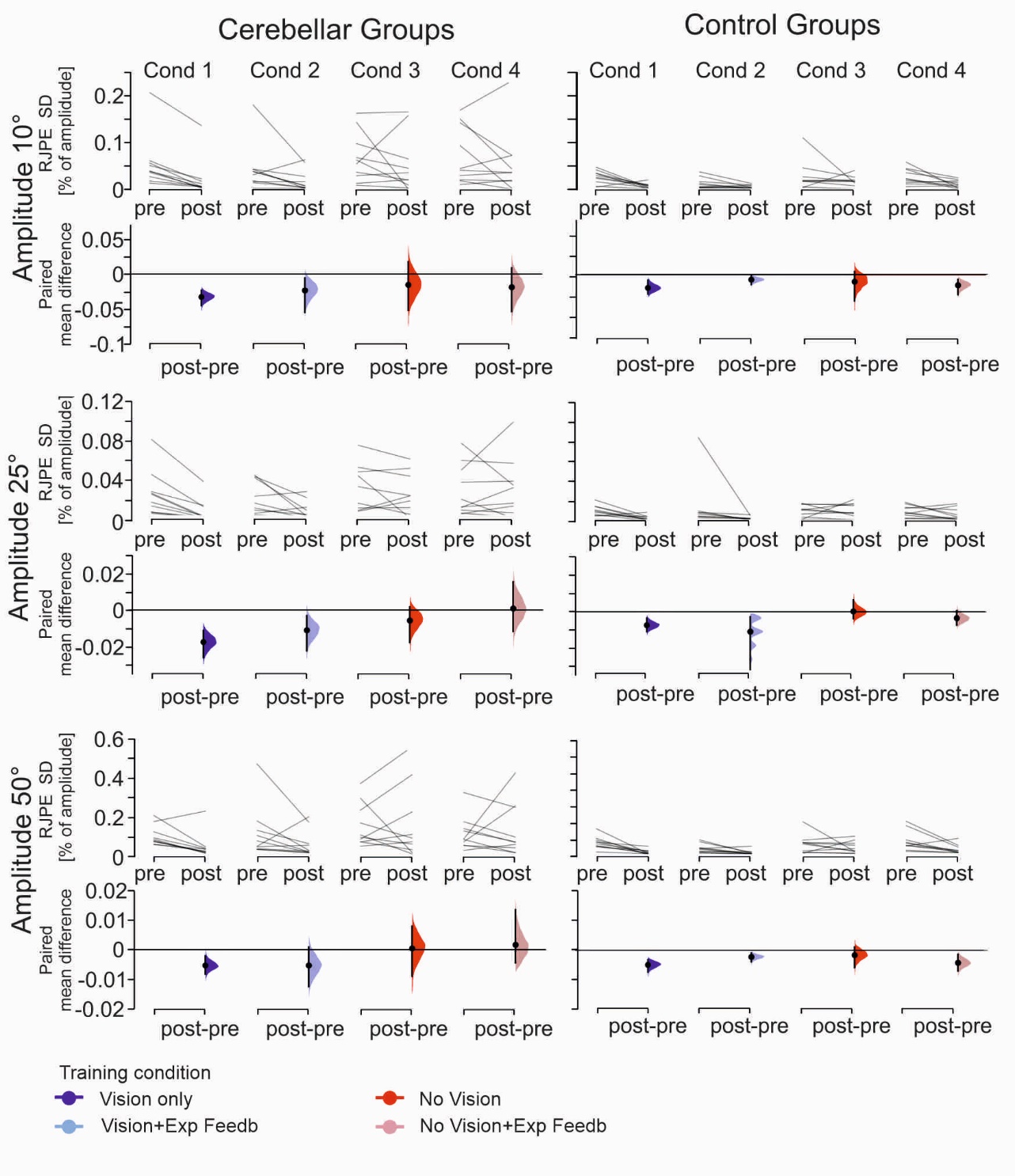
**

**Fig. 3-1**

Multi-paired estimation plots displaying individual data points and effect sizes comparing RJPE SD between the pre- and post-training (post) for movement amplitudes 10°, 25° and 50° in cerebellar patients and controls. Thin lines in upper panels represent individual data points (pre vs. post) of each participant. Lower panels show effects sizes. Black dots represent mean differences between pre- and post-training assessment in each subgroup and error bars 95% confidence intervals (CI). 95% CI are calculated by bootstrap resampling (Ho et al., 2019). Filled curves represent the bootstrap sampling distribution of the observed data. Multi-paired estimation plots were generated using the web-application of DABEST ("data analysis with bootstrap-coupled estimation”; <http://www.estimationstats.com/>).

**
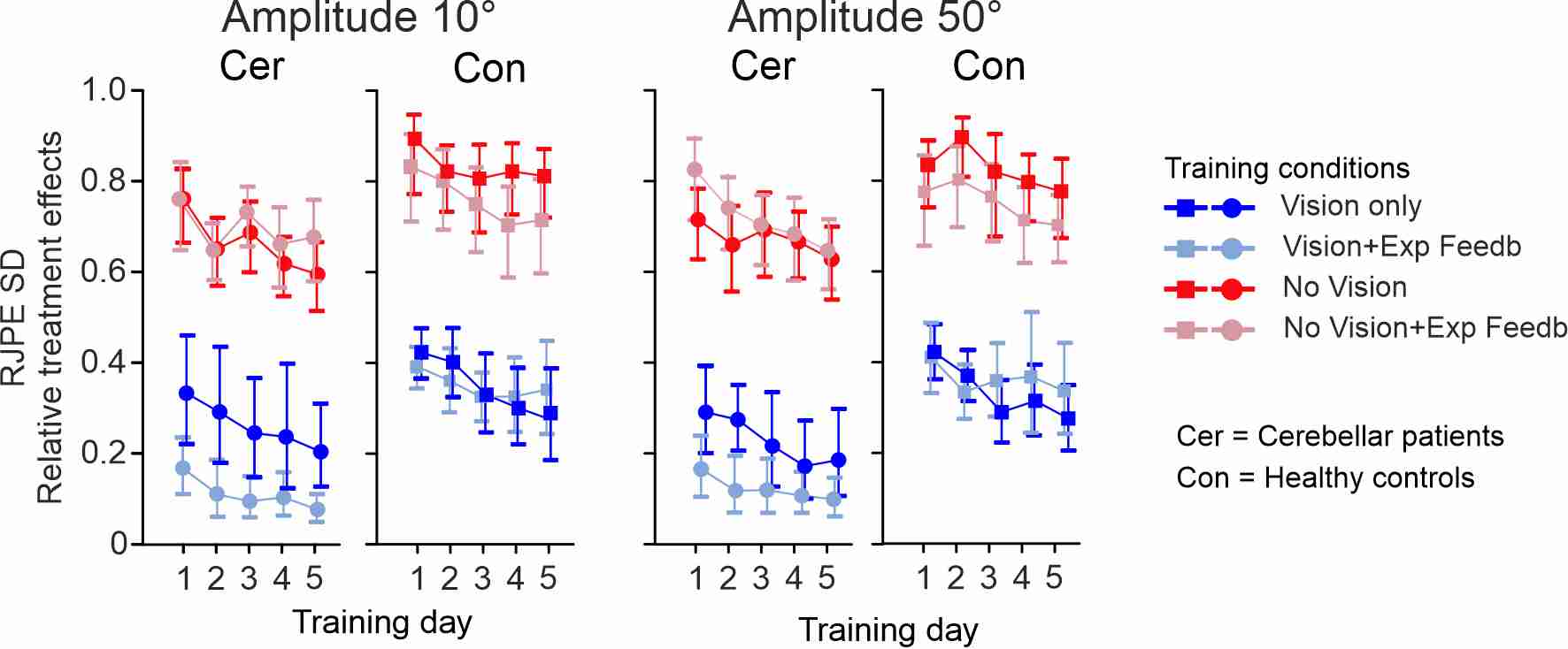
**

**Fig. 4-1**

Relative treatment effects for RJPE SD across the five training days considering movement amplitudes 10° and 50° in cerebellar patients (Cer) and controls (Con). Median relative treatment effects and 95% confidence intervals are shown comparing the four training conditions

**Supplementary Tables**

**Table 3-1**

Mean peak velocity (V_max_) – pre-training/post-training. V_max_ is expressed in degree per second (°/s). Group means and standard deviations comparing pre-training (Pre) and post-training (Post) assessments in **A)** cerebellar patients and **B)** controls of the three movement amplitudes (10°, 25°, 50°) in the four training conditions (cond): 1 = **Vision Only;** 2 = **Vision + Exp Feedb;** 3 = **No Vision;** 4 = **No Vision + Exp Feedb**

| **A)** | **Cerebellar patients** | | | | | | | | | | | |
| --- | --- | --- | --- | --- | --- | --- | --- | --- | --- | --- | --- | --- |
|  | Amplitude 10° | | | | Amplitude 25° | | | | Amplitude 50° | | | |
| Cond | 1 | 2 | 3 | 4 | 1 | 2 | 3 | 4 | 1 | 2 | 3 | 4 |
| Pre | 31.61  ± 9.74 | 28.72  ± 5.24 | 32.30  ± 8.79 | 32.47  ± 11.2 | 57.31  ± 12.3 | 54.22  ± 13.6 | 56.31  ± 11.4 | 57.35  ± 13.8 | 88.99  ± 21.6 | 91.40  ± 26.0 | 91.68  ± 21.4 | 93.44  ± 22.7 |
| Post | 25.11  ± 4.56 | 25.31  ± 5.79 | 28.17  ± 6.87 | 28.34  ± 4.82 | 46.72  ± 6.77 | 49.00  ± 13.0 | 52.28  ± 12.6 | 55.55  ± 11.0 | 78.41  ± 13.1 | 81.04  ± 23.2 | 84.85  ± 18.3 | 91.61  ± 20.3 |
| **B)** | **Controls** | | | | | | | | | | | |
|  | Amplitude 10° | | | | Amplitude 25° | | | | Amplitude 50° | | | |
| Cond | 1 | 2 | 3 | 4 | 1 | 2 | 3 | 4 | 1 | 2 | 3 | 4 |
| Pre | 27.98 ± 4.51 | 29.12 ± 5.29 | 29.04  ± 4.90 | 27.81 ± 3.42 | 52.21 ± 8.71 | 56.66 ± 8.68 | 55.14 ± 7.12 | 51.35  ± 7.21 | 88.59 ± 17.0 | 92.72 ± 11.7 | 91.15 ± 11.8 | 88.24 ± 12.5 |
| Post | 24.71 ± 4.13 | 25.74 ± 4.55 | 26.21 ± 2.79 | 26.48  ± 3.86 | 48.08 ± 9.15 | 49.77 ± 7.73 | 51.58 ± 5.50 | 51.00  ± 8.19 | 84.68 ± 15.1 | 86.70 ± 15.5 | 89.91 ± 11.5 | 88.27 ± 13.8 |

**Table 3-2**

Mean peak velocity (V_max_) – pre-training vs. post-training. Summary of statistical results considering pre-training and post-training assessments. Nonparametric rank-based ANOVA-type tests for factorial longitudinal data were applied. Degrees of freedom were adjusted in case variances differed.

| **Amplitude** | **Effect** | **Mean peak velocity (V_max_)** |
| --- | --- | --- |
| **10°** | Group | F(1,64.7)=0.90; p=0.3452 |
|  | Training condition | F(2.98,64.7)=0.97; p=0.4110 |
|  | Group x Training condition | F(2.98,64.7)=0.36; p=0.7834 |
|  | Time (pre vs. post) | F(1,59.8)=24.09; p<.0001 |
|  | Group x Time | F(1,59.8)=0.00; p=0.9487 |
|  | Training condition x Time | F(2.84,59.8)=0.88; p=0.4529 |
|  | Group x Condition x Time | F(2.84,59.8)=0.03; p=0.9923 |
| **25°** | Group | F(1,64)=0.12; p=0.7304 |
|  | Training condition | F(2.99,64)=0.81; p=0.4932 |
|  | Group x Training condition | F(2.99,64)=0.68; p=0.5679 |
|  | Time (pre vs. post) | F(1,66.7)=17.44; p<.0001 |
|  | Group x Time | F(1,66.7)=0.00; p=0.9720 |
|  | Training condition x Time | F(2.92,66.7)=2.03; p=0.1196 |
|  | Group x Condition x Time | F(2.92,66.7)=0.31; p=0.8157 |
| **50°** | Group | F(1,62.4)=0.74; p=0.3926 |
|  | Training condition | F(2.99,62.4)=0.67; p=0.5727 |
|  | Group x Training condition | F(2.99,62.4)=0.23; p=0.8733 |
|  | Time (pre vs. post) | F(1,57.1)=5.85; p=0.0188 |
|  | Group x Time | F(1,57.1)=0.80; p=0.3746 |
|  | Training condition x Time | F(2.65,57.1)=0.61; p=0.5932 |
|  | Group x Condition x Time | F(2.65,57.1)=0.17; p=0.8954 |

**Table 4-1**

RJPE mean (RJPE M) – five training days. RJPE M is expressed as percentage of the movement amplitude. Group means and standard deviations across the five training days in **A)** cerebellar patients and **B)** controls of the two movement amplitudes (10°, 50°) in the four training conditions (cond): 1 = **Vision Only;** 2 = **Vision + Exp Feedb;** 3 = **No Vision;** 4 = **No Vision + Exp Feedb**

| **A)** | **Cerebellar group** | | | | | | | |
| --- | --- | --- | --- | --- | --- | --- | --- | --- |
|  | Amplitude 10° | | | | Amplitude 50° | | | |
| Training days/Cond | 1 | 2 | 3 | 4 | 1 | 2 | 3 | 4 |
| Day 1 | 4.79  ± 2.76 | 4.82  ± 2.79 | 18.71  ± 5.29 | 16.29  ± 5.16 | 0.98  ± 0.41 | 0.95  ± 0.59 | 4.54  ± 1.32 | 4.26  ± 2.06 |
| Day 2 | 4.00  ± 1.99 | 3.63  ± 1.95 | 17.23  ± 5.70 | 13.98  ± 3.85 | 0.82 ± 0.37 | 0.82  ± 0.48 | 4.71 ± 2.03 | 3.92  ± 0.95 |
| Day 3 | 2.96  ± 1.58 | 3.81  ± 1.77 | 15.53  ± 4.42 | 13.46  ± 3.48 | 0.57  ± 0.27 | 0.88  ± 0.60 | 4.75  ± 2.56 | 3.65  ± 0.91 |
| Day 4 | 2.79  ± 1.58 | 3.68  ± 2.38 | 17.28  ± 3.76 | 13.49  ± 3.98 | 0.54  ± 0.31 | 1.02 ± 0.94 | 4.32 ± 1.37 | 3.26 ± 0.88 |
| Day 5 | 3.06  ± 3.58 | 4.17 ± 2.83 | 16.24  ± 3.89 | 13.25 ± 3.67 | 0.53 ± 0.31 | 0.89 ± 0.60 | 3.83 ± 1.35 | 3.42 ± 0.75 |
| **B)** | **Control group** | | | | | | | |
|  | Amplitude 10° | | | | Amplitude 50° | | | |
| Training days/Cond | 1 | 2 | 3 | 4 | 1 | 2 | 3 | 4 |
| Day 1 | 3.70  ± 4.31 | 1.75  ± 0.85 | 13.47  ± 4.04 | 14.01  ± 3.09 | 0.69  ± 0.85 | 0.34  ± 0.19 | 3.44  ± 0.63 | 3.75  ± 0.67 |
| Day 2 | 3.90  ± 5.26 | 1.27  ± 0.89 | 11.48  ± 2.70 | 11.80  ± 2.69 | 0.63  ± 0.77 | 0.32  ± 0.20 | 3.00  ± 1.18 | 3.82  ± 0.90 |
| Day 3 | 3.17  ± 4.23 | 1.21  ± 0.79 | 13.32  ± 2.32 | 11.96  ± 2.36 | 0.65  ± 0.86 | 0.32  ± 0.16 | 3.07  ± 0.92 | 3.29  ± 0.69 |
| Day 4 | 3.17  ± 3.76 | 1.14  ± 0.64 | 11.40  ± 1.76 | 11.61  ± 2.00 | 0.60  ± 0.77 | 0.32  ± 0.08 | 3.08  ± 0.78 | 3.22  ± 0.99 |
| Day 5 | 2.94  ± 3.56 | 0.95  ± 0.57 | 11.75  ± 1.90 | 11.31  ± 3.35 | 0.49  ± 0.57 | 0.23  ± 0.06 | 3.03  ± 0.86 | 3.06  ± 1.03 |

**Table 4-2**

RJPE standard deviation (RJPE SD) – five training days. RJPE SD is expressed as percentage of the movement amplitude. Group means and standard deviations across the five training days in **A)** cerebellar patients and **B)** controls of the two movement amplitudes (10°, 50°) in the four training conditions (cond): 1 = **Vision Only;** 2 = **Vision + Exp Feedb;** 3 = **No Vision;** 4 = **No Vision + Exp Feedb**

| **A)** | **Cerebellar group** | | | | | | | |
| --- | --- | --- | --- | --- | --- | --- | --- | --- |
|  | Amplitude 10° | | | | Amplitude 50° | | | |
| Training days/Cond | 1 | 2 | 3 | 4 | 1 | 2 | 3 | 4 |
| Day 1 | 3.78  ± 2.40 | 3.33  ± 1.78 | 15.89  ± 4.98 | 13.96  ± 5.51 | 0.98  ± 0.69 | 0.88  ± 0.77 | 3.48  ± 1.03 | 3.32  ± 1.68 |
| Day 2 | 3.37  ± 2.54 | 2.95  ± 2.16 | 13.86  ± 6.33 | 11.87  ± 3.36 | 0.69  ± 0.43 | 0.61  ± 0.48 | 3.79  ± 0.83 | 3.22  ± 0.92 |
| Day 3 | 2.79  ± 2.55 | 2.54  ± 1.59 | 13.18  ± 5.24 | 11.22  ± 3.98 | 0.48  ± 0.34 | 0.68  ± 0.70 | 3.60  ± 1.46 | 2.96  ± 0.83 |
| Day 4 | 2.35  ± 2.31 | 2.73  ± 2.15 | 13.04  ± 4.27 | 10.22  ± 3.53 | 0.56  ± 0.50 | 0.84  ± 1.27 | 3.11  ± 0.68 | 2.68  ± 0.84 |
| Day 5 | 2.26  ± 2.44 | 3.01  ± 3.01 | 12.55  ± 3.78 | 10.65  ± 3.80 | 0.47  ± 0.40 | 0.63  ± 0.81 | 3.04  ± 0.84 | 2.58  ± 0.67 |
| **B)** | **Control group** | | | | | | | |
|  | Amplitude 10° | | | | Amplitude 50° | | | |
| Training days/Cond | 1 | 2 | 3 | 4 | 1 | 2 | 3 | 4 |
| Day 1 | 2.88  ± 3.48 | 1.09  ± 0.70 | 10.86  ± 2.55 | 11.39  ± 3.56 | 0.52  ± 0.54 | 0.25  ± 0.17 | 2.71  ± 0.65 | 3.36  ± 0.91 |
| Day 2 | 2.61  ± 4.05 | 0.79  ± 0.64 | 8.92  ± 1.67 | 8.89  ± 1.31 | 0.48  ± 0.56 | 0.20  ± 0.13 | 2.33  ± 0.87 | 2.83  ± 0.71 |
| Day 3 | 2.03  ± 2.92 | 0.71  ± 0.51 | 9.60  ± 1.96 | 10.33  ± 2.43 | 0.45  ± 0.72 | 0.19  ± 0.11 | 2.58  ± 0.85 | 2.60  ± 0.66 |
| Day 4 | 2.21  ± 3.69 | 0.74  ± 0.55 | 8.42  ± 1.33 | 9.28  ± 2.14 | 0.36  ± 0.53 | 0.18  ± 0.08 | 2.43  ± 0.60 | 2.49  ± 0.83 |
| Day 5 | 1.72  ± 2.35 | 0.59  ± 0.29 | 8.24  ± 1.74 | 9.53  ± 2.58 | 0.41  ± 0.67 | 0.17  ± 0.07 | 2.22  ± 0.64 | 2.33  ± 0.66 |

**Table 4-3**

Mean peak velocity (V_max_) – five training days. V_max_ is expressed in degree per second (°/s). Group means and standard deviations across the five training days in **A)** cerebellar patients and **B)** controls of the two movement amplitudes (10°, 50°) in the four training conditions (cond): 1 = **Vision Only;** 2 = **Vision + Exp Feedb;** 3 = **No Vision;** 4 = **No Vision + Exp Feedb**

| **A)** | **Cerebellar group** | | | | | | | |
| --- | --- | --- | --- | --- | --- | --- | --- | --- |
|  | Amplitude 10° | | | | Amplitude 50° | | | |
| Training days/Cond | 1 | 2 | 3 | 4 | 1 | 2 | 3 | 4 |
| Day 1 | 28.33 ± 8.50 | 28.91 ± 7.79 | 26.37 ± 4.08 | 26.78 ± 6.98 | 96.45 ± 30.8 | 94.87 ± 30.6 | 88.10 ± 9.31 | 94.63 ± 20.7 |
| Day 2 | 26.12 ± 5.68 | 27.10 ± 7.23 | 24.80 ± 3.93 | 25.28 ± 4.36 | 86.70 ± 19.0 | 87.00 ± 23.5 | 83.85 ± 11.6 | 87.52 ± 15.4 |
| Day 3 | 24.12 ± 4.51 | 27.29 ± 9.42 | 24.61 ± 4.27 | 24.83 ± 4.36 | 84.63 ± 18.3 | 89.14 ± 26.5 | 85.67 ± 12.0 | 86.93 ± 19.0 |
| Day 4 | 24.34 ± 5.16 | 25.60 ± 7.08 | 24.53 ± 3.41 | 24.67 ± 7.09 | 81.99 ± 19.7 | 85.22 ± 26.1 | 81.87 ± 11.1 | 87.55 ± 22.3 |
| Day 5 | 23.83 ± 4.23 | 24.61 ± 7.13 | 24.65 ± 3.00 | 25.13 ± 6.49 | 79.86 ± 15.1 | 83.02 ± 24.7 | 82.55 ± 13.9 | 87.78 ± 22.9 |
| **B)** | **Control group** | | | | | | | |
|  | Amplitude 10° | | | | Amplitude 50° | | | |
| Training days/Cond | 1 | 2 | 3 | 4 | 1 | 2 | 3 | 4 |
| Day 1 | 25.61 ± 3.11 | 26.79 ± 3.31 | 24.24 ± 4.36 | 27.12 ± 5.02 | 90.71 ± 15.6 | 97.65 ± 11.4 | 93.69 ± 12.2 | 91.16 ± 22.9 |
| Day 2 | 24.30 ± 3.30 | 25.61 ± 3.36 | 24.95 ± 2.61 | 25.47 ± 4.74 | 86.20 ± 15.6 | 92.33 ± 15.3 | 94.22 ± 6.82 | 93.86 ± 16.3 |
| Day 3 | 24.73 ± 2.40 | 25.11 ± 3.71 | 25.25 ± 3.24 | 25.33 ± 5.98 | 86.76 ± 12.6 | 93.09 ± 15.2 | 90.38 ± 7.84 | 92.22 ± 15.3 |
| Day 4 | 23.46 ± 2.89 | 24.76 ± 3.99 | 25.28 ± 3.56 | 26.14 ± 6.71 | 82.11 ± 9.79 | 86.80 ± 15.6 | 95.56 ± 10.5 | 93.99 ± 20.4 |
| Day 5 | 23.90 ± 3.58 | 24.82 ± 3.69 | 26.15 ± 3.75 | 25.04 ± 5.76 | 82.69 ± 19.9 | 86.09 ± 13.9 | 94.72 ± 11.0 | 96.60 ± 20.4 |

**Table 4-4**

Mean peak velocity (V_max_) – five training days. Summary of statistical results considering the five training days. Nonparametric rank-based ANOVA-type tests for factorial longitudinal data were applied. Degrees of freedom were adjusted in case variances differed.

| **Amplitude** | **Effect** | **Mean peak velocity (V_max_)** |
| --- | --- | --- |
| **10°** | Group | F(1,61.7)= 0.01; p=0.9093 |
|  | Training condition | F(2.89,61.7)=0.12; p=0.9414 |
|  | Group x Training condition | F(2.89,61.7)=0.06; p=0.9764 |
|  | Time (training days 1-5) | F(2.6,124)=7.40; p=0.0003 |
|  | Group x Time | F(2.6,124)=1.08; p=0.3556 |
|  | Training condition x Time | F(6.62,124)=1.05; p=0.3973 |
|  | Group x Condition x Time | F(6.62,124)=0.83; p=0.5602 |
| **50°** | Group | F(1,58)=2.29; p=0.1355 |
|  | Training condition | F(2.76,58)=0.56; p=0.6294 |
|  | Group x Training condition | F(2.76,58)=0.40; p=0.7362 |
|  | Time (training days 1-5) | F(2.5,125)=8.87; p<.0001 |
|  | Group x Time | F(2.5,125)=1.29; p=0.2804 |
|  | Training condition x Time | F(6.86,125)=1.29; p=0.2635 |
|  | Group x Condition x Time | F(6.86,125)=1.44; p=0.1966 |
